# Efficient prediction of reaction paths through molecular graph and reaction network analysis†
†Electronic supplementary information (ESI) available: Detailed information on reaction networks and pathways for two example reactions, Cartesian coordinates of molecules in the reaction networks obtained at the DFT level for the hydroformylation example, and conformers and isomers of the intermediates in the Heck–Breslow mechanism. See DOI: 10.1039/c7sc03628k


**DOI:** 10.1039/c7sc03628k

**Published:** 2017-12-12

**Authors:** Yeonjoon Kim, Jin Woo Kim, Zeehyo Kim, Woo Youn Kim

**Affiliations:** a Department of Chemistry , KAIST , 291 Daehak-ro, Yuseong-gu , Daejeon 34141 , Korea . Email: wooyoun@kaist.ac.kr

## Abstract

Despite remarkable advances in computational chemistry, prediction of reaction mechanisms is still challenging, because investigating all possible reaction pathways is computationally prohibitive due to the high complexity of chemical space. A feasible strategy for efficient prediction is to utilize chemical heuristics. Here, we propose a novel approach to rapidly search reaction paths in a fully automated fashion by combining chemical theory and heuristics. A key idea of our method is to extract a minimal reaction network composed of only favorable reaction pathways from the complex chemical space through molecular graph and reaction network analysis. This can be done very efficiently by exploring the routes connecting reactants and products with minimum dissociation and formation of bonds. Finally, the resulting minimal network is subjected to quantum chemical calculations to determine kinetically the most favorable reaction path at the predictable accuracy. As example studies, our method was able to successfully find the accepted mechanisms of Claisen ester condensation and cobalt-catalyzed hydroformylation reactions.

## Abstract 2

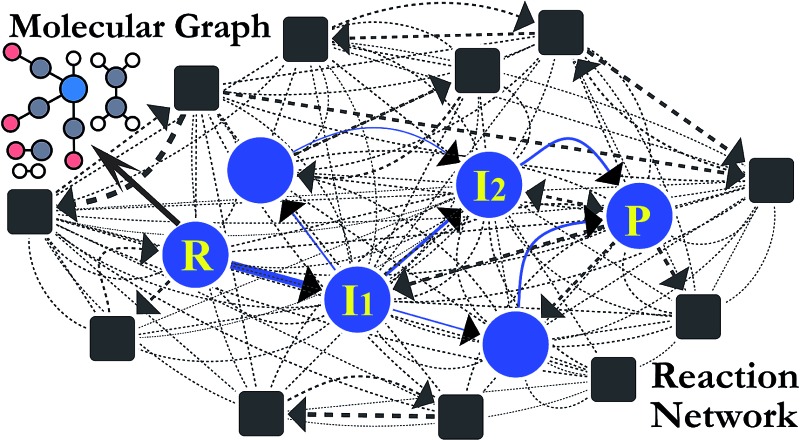
A minimal subnetwork is extracted from a very complex full network upon exploring the reaction pathways connecting reactants and products with minimum dissociation and formation of chemical bonds. Such a process reduces computational cost and correctly predicts the pathway for two representative reactions.

## Introduction

Computational chemistry is a powerful approach for the mechanistic study of chemical reactions, because it can offer deep insight on reaction mechanisms at the atomistic level.^1^–^3^ Remarkable advances in quantum chemistry especially with the rise of density functional theory (DFT) have provided a handful tool to obtain energy profiles of reaction paths for verifying experimentally and/or intuitively proposed mechanisms. However, prediction of chemical reactions is still challenging because exploring all possible paths on potential energy surface (PES) is intractable due to its high complexity.^4^–^6^

Substantial efforts have been devoted to developing automated exploration methods of reaction paths on the PES.^7^–^16^ Maeda and coworkers developed so-called anharmonic downward distortion following method for global exploration of isomerization paths for a single molecule.^7^,^8^ They also proposed the artificial force-induced reaction method that finds a reaction path through accelerated chemical reactions with an artificial force.^9^–^11^ Local minima-sampling methods such as basin-hopping Monte-Carlo and minima hopping algorithms can be used to find appropriate reaction intermediates.^6^,^17^–^20^ We also reported a graph-theoretic approach combined with the basin-hopping Monte-Carlo algorithm.^21^ Heuristic rules combined with quantum chemistry were utilized for the efficient generation of intermediates. For instance, Bergler and coworkers discovered new intermediates through the structural relaxation of numerous reactive complexes prepared according to their reactivity.^22^,^23^ This approach has been used for the automated exploration of reaction paths with the quantification of uncertainties in solving rate equations.^24^ An efficient method combining a transition state search using accelerated chemical dynamics with kinetic Monte-Carlo simulations has also been devised for the kinetic study of organometallic catalysis.^25^,^26^ However, those methods inevitably require large computational costs, since every movement of molecules on the PES entails quantum chemical calculations.

**Fig. 1:**
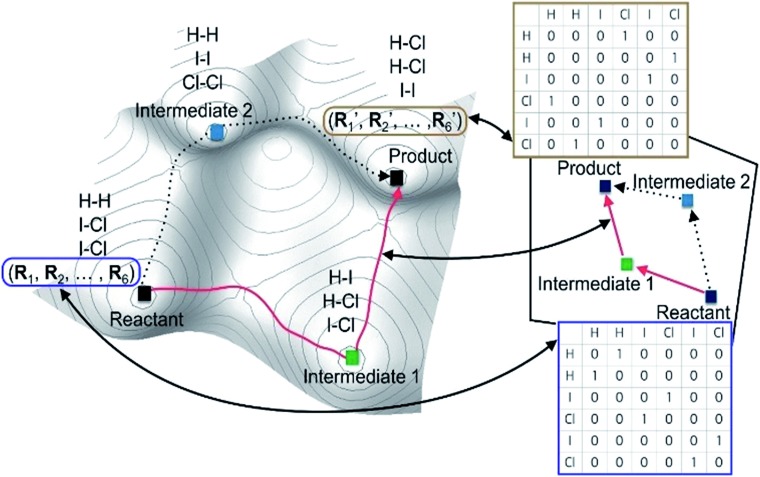
Correspondence between a real potential energy surface and a hypothetical chemical space represented with molecular graphs for the reaction in eqn (1) as an example.

Alternatively, chemical reactions can be described by the successive change in the chemical bonds of reactive molecules. Stable molecular structures as local minima on the PES can be mapped to molecular graphs, as illustrated in Fig. 1. In this point of view, chemical reactions may be equivalently described by the successive conversion of a reactant molecular graph into isomeric graphs. In fact, this kind of graph-theoretic approaches has attracted great attention in the past for computer-assisted mechanistic study thanks to their efficient algorithm accelerated by heuristic rules.^27^–^45^

Most graph-theoretic methods adopt one of the following three steps or a combination of them. First, the combinatorial enumeration of molecular graphs generates a set of molecules that can be made from the reactants. For example, if a table of reaction graphs is available, Pólya’s theorem offers an analytical enumeration technique considering permutations due to molecular symmetry.^46^,^47^ A constructive enumeration algorithm can also be used to find a complete list of graphs.^48^–^50^ Second, molecules obtained from the resulting graphs are linked with each other to make hypothetical elementary reactions, leading to a chemical reaction network. Finally, the network is analyzed to determine kinetically favorable reaction mechanisms. In each procedure, they adopt heuristic rules based on chemical concepts and databases, which limits their applicability and reliability. In 2017, Segler and Waller proposed a data-driven model to improve such rule-based methods by predicting the reactivity of molecules based on the complete published knowledge, but its use is limited to binary reactions.^51^

More recently, complementary approaches are being actively developed to exploit the advantages of both graph-theoretic and quantum chemical methods.^22^,^23^,^52^–^61^ Molecular graphs are used to generate hypothetical reaction intermediates. To find kinetically feasible elementary reaction steps, the activation energy between two intermediates is explicitly calculated using conventional methods such as the nudged elastic band^52^,^53^ and eigenvector-following with freezing^22^,^54^ and growing^55^–^58^ string methods. Single-ended algorithms such as Berny optimization and intrinsic reaction coordinate calculations^62^–^64^ can be employed for further improvements. To consider multiple paths, Habershon devised a novel constrained molecular dynamics using a model Hamiltonian.^52^,^53^ Hammond’s postulate^65^ was also used for efficiency.^59^,^60^ These methods aim to automatically discover reaction mechanisms from a single input molecular structure with minimal human efforts and were successfully applied to several organic reactions. However, molecular graph enumeration results in a huge number of intermediates and hypothetical elementary reactions due to combinatorial explosion. As a result, calculating transition states for every elementary reaction is a computational bottleneck. Therefore, it is critical to remove chemically irrelevant hypothetical elementary reactions in an efficient way for the success of such automated approaches.

Here we propose a fast prediction method of reaction paths through molecular graph and reaction network analysis. Our method adopts some idea of the aforementioned methods such as molecular graph enumeration,^52^–^61^ but also introduces new fascinating features; a key distinctive one is to efficiently extract the minimal subnetwork from a complex full network. This can be done by exploring multiple reaction paths connecting reactants and products with minimum dissociation and formation of chemical bonds using a graph-theoretic method. Another important feature of our method is its wide applicability. This is because the method is able to explore in principle all possible reaction routes by considering all combinations of chemical bond formation and dissociation, which is typically intractable. To make it computationally efficient, we devised *de novo* protocols to rule out many unimportant reaction routes and intermediates according to general chemical rules. As a result, fast searching for most plausible reaction paths is feasible within an hour on a single workstation. The resultant paths can be verified with further refinements using quantum chemical methods. This first-screening and then-verifying strategy minimizes expensive computational parts, which is critical to achieve both predictive power and efficiency. In what follows, we first explain the details of the proposed method. Then, to demonstrate its reliability and efficiency, we provide two example studies: a well-known organic reaction and a simple organometallic reaction. Finally, we conclude with a summary and outlook for future works.

## Methods

Most graph-theoretic approaches are based on the following concept; chemical reactions can be described by changing molecular graphs according to heuristic rules. This implicitly assumes that there exists a hypothetical chemical space, in which molecular structures are expressed with graphs. Fig. 1 schematically illustrates the relation between a real PES and the corresponding chemical space. The left figure shows the PES of the following reaction (eqn (1)) as an example.

1 2ICl + H_2_ → HI + HCl + ICl → 2HCl + I_2_.

Local minima on the PES include reactants, intermediates, and products. The chemical reaction occurs along the minimum energy path between the reactants and the products, as indicated by the red line in Fig. 1. The same chemistry can be described in the hypothetical chemical space (the right side of Fig. 1). The local minima can be mapped to molecular graphs in the chemical space. An elementary reaction between two intermediates corresponds to an edge linking two graphs. The length of each edge is related to the rate constant of the corresponding elementary reaction.

A key advantage of using the hypothetical chemical space is that all possible molecular graphs, each of which corresponds to a stable molecular structure, can efficiently be generated by the combinatorial enumeration of an input graph, resulting in an extensive set of reaction intermediates without resorting to quantum chemical calculations. This fascinating feature enables us to avoid huge computational costs required by direct searching methods on PES. However, the hypothetical chemical space may not be complete to encompass the entire PES; for instance, conformational isomers are mapped to an identical molecular graph. Some of such problems can be resolved by using additional quantum chemical methods. Hence, this approach can be applied to a wide range of chemical reactions.

**Fig. 2:**
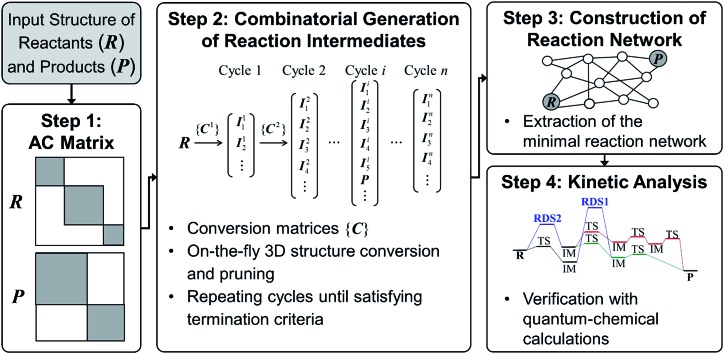
Flowchart of our graph-based method for fast prediction of reaction paths.

Fig. 2 shows the flowchart of our method. As in previous works,^52^–^61^ we use molecular graphs expressed specifically with an atom connectivity (AC) matrix to represent molecular structures. Namely the bond-electron matrix can also be used as an alternative to the AC matrix.^27^,^28^,^54^ It is different from the AC matrix in its diagonal elements containing the number of valence electrons that do not participate in chemical bonds. Therefore, an electron-pushing model mimicking the language of organic chemistry can be utilized for matrix enumeration. However, those two models are equivalent with one another in a sense that one of them can be converted to the other by a graph-theoretic analysis.^66^,^67^

Since we aim to find a reaction path from given reactants (***R***) to designated products (***P***), both ***R*** and ***P*** structures are given as an input and converted to the corresponding AC matrices in Step 1. As is indicated by the shaded color, the reactant and product matrices may contain a few block matrices, each of which denotes a constituent molecule of ***R*** and ***P***, respectively. In Step 2, a number of AC matrices are generated by consecutively applying a set of conversion matrices ({***C***}) to ***R*** until satisfying predefined termination criteria, which corresponds to the combinatorial enumeration of molecular graphs. In Step 3, a reaction network is constructed by calculating the length of edges between the AC matrices. Finally, the reaction network is analyzed to determine kinetically favorable reaction paths. Steps 1 and 2 are similar to other methods.^29^ In Step 3, however, we introduce a novel graph-theoretic method to extract a subnetwork including an essential part directly relevant to reaction mechanism from the full network. In Step 4, we perform transition state calculations for the extracted minimal reaction network to determine kinetically the most favorable reaction path. In what follows, we explain Steps 2–4 more in detail.

### 1. Step 2: combinatorial generation of reaction intermediates

#### Enumeration of AC matrices

For the combinatorial sampling of intermediates using molecular graphs, we start with the AC matrix of reactants as illustrated in Fig. 2. In the first cycle, a set of conversion matrices ({***C***^1^}) is applied to ***R*** to generate a set of intermediates ({***I***^1^}) through dissociation and formation of bonds; ***R*** + ***C***_*j*_^1^ = ***I***_*j*_^1^. In the second cycle, a new set of conversion matrices ({***C***^2^}) is constructed for each of {***I***^1^} and is applied to it to obtain new intermediates; ***I***_*j*_^1^ + ***C***_*j*_^2^ = ***I***_*j*_^2^. This process is repeated until predefined termination criteria are satisfied. The conversion matrix ***C*** for a given intermediate ***I*** is constructed as follows. Elements of ***C*** consist of 1, –1, and 0, which correspond to the formation, cleavage, and no change of chemical bonds, respectively. To take into account all possible combinations of chemically allowed matrix elements, we use the following rules:2
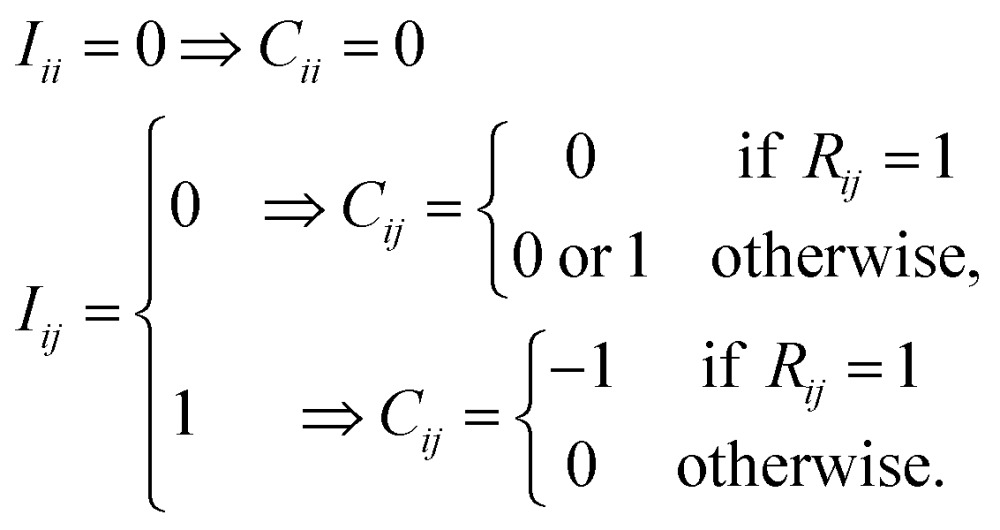



All diagonal elements are zero. If an off-diagonal element of ***I*** is zero (*I*_*ij*_ = 0), the corresponding element of ***C*** can be either 0 or 1. If the same row-column element of ***R*** is 1 (*R*_*ij*_ = 1), *C*_*ij*_ = 0 because *I*_*ij*_ = 0 means that the chemical bond between atoms *i* and *j* in reactants has been broken in a previous cycle. This condition is necessary to prevent from generating AC matrices appeared in previous cycles once again. Otherwise, *C*_*ij*_ = 0 or 1. Similarly, for *I*_*ij*_ = 1, *C*_*ij*_ = –1, if *R*_*ij*_ = 1. Otherwise, *C*_*ij*_ = 0.

The above rules will produce all possible conversion matrices for each ***I***. However, it is inefficient for a large matrix. Various user-defined constraints may be helpful to reduce computational costs. At the same time, excessive constraints may provoke biased results. To compromise between efficiency and reliability, we apply only the following two constraints. The maximum number of bond formations and dissociations at each elementary reaction is limited to two, respectively, *i.e.*,3
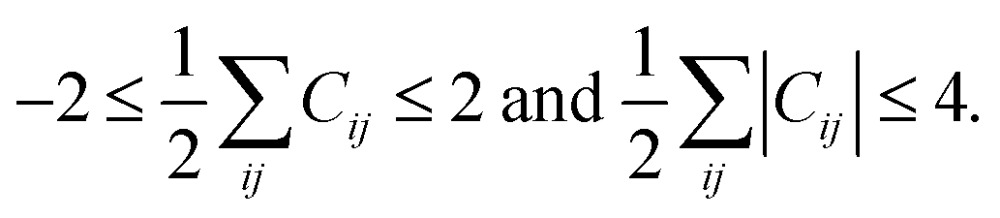



The same constraint was imposed in other works.^54^,^55^ Only unimolecular and bimolecular reactions at each elementary reaction are allowed. This constraint is problematic for termolecular reactions, but it still encompasses most organic reactions. These two constraints can be controlled as input variables. It is also important to delete permutational isomers (or isomorphic copies) produced by the combinatorial enumeration. They can be discriminated by investigating the eigenvalues of the *alternative* Coulomb matrix of each AC matrix, which is modified from the original Coulomb matrix to detect graph isomorphism.^21^ This conversion cycle is continued to find a number of intermediates including products and finally terminated if no new matrix is generated. Other termination conditions such as the maximum number of cycles can also be imposed.

#### On-the-fly 3D structure conversion and pruning

AC matrices at each cycle are subjected to on-the-fly 3D structure conversion and pruning. In our previous work, we proposed a reliable method that sequentially converts a given AC matrix to a bond order matrix, then to a SMILES code, and finally to a 3D geometry.^66^,^67^ The reliability of this process has been proved by successfully applying it to 10 000 organic molecules randomly chosen from the PubChem database.^66^ While we refer to ref. 66 for the technical details of the method, we here provide its overall procedure briefly. At each step, we screen out inappropriate molecular structures as follows. Information on the atomic valence and formal charge of each atom can be deduced by transforming AC matrices to bond order matrices. Molecules having atoms with inappropriate atomic valence or formal charge are discarded. In the SMILES conversion step, those having an inappropriate number of rings for a given reaction are removed. The remaining SMILES codes are then converted to 3D geometries, which are subsequently optimized by using conventional methods with desirable accuracy.

Our structure conversion method also yields all stereoisomers of organic molecules that can be constructed from a given AC matrix; *cis*–*trans* isomers and enantiomers can be specified explicitly by SMILES, and they are readily converted to 3D geometries, as explained in ref. 66. To find all possible conformers sharing an identical AC, we perform additional basin-hopping Monte-Carlo samplings with bond constraints to prevent from breaking the AC, as explained in ref. 21. However, conformers and stereoisomers of a metal complex cannot be specified by SMILES. Therefore, we developed a new method combining the bond constraint basin-hopping Monte-Carlo method with force field calculations. At each basin-hopping sampling cycle, the relative positions of ligands with respect to the metal center are distributed randomly, and this new structure is relaxed using force field calculations with fixed AC and bond orders. After finishing the combinatorial enumeration, molecules with energy higher than a threshold are screened out. The threshold energy is defined as the sum of reactant energy and a given tolerance value (*E*_tol_).

#### Introduction to active atoms

**Fig. 3:**
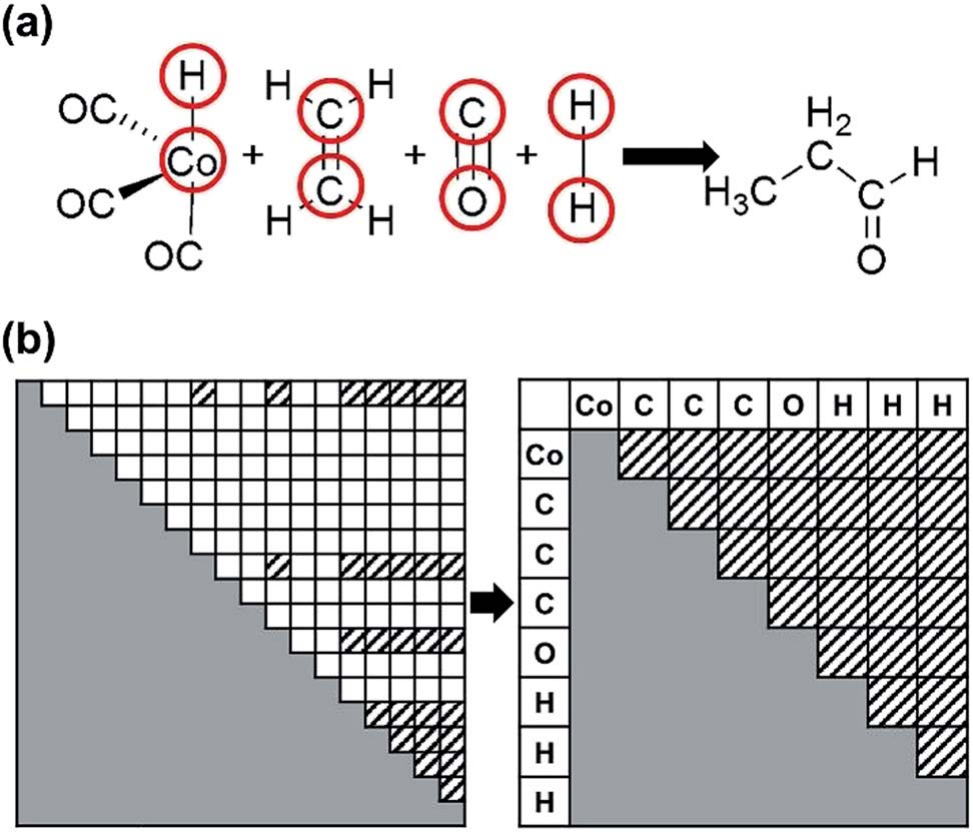
(a) The reactant and product molecules for the cobalt-catalyzed hydroformylation reaction. The red circles indicate active atoms. (b) Construction of an active-atom connectivity matrix from an all-atom connectivity matrix. Matrix elements filled with slashes indicate bonds between active atoms.

Although the combinatorial sampling with the above rules is very comprehensive, it is demanding to deal with a large number of atoms due to combinatorial explosion. Fortunately, we note that, in most chemical reactions, only a few atoms regarded as reaction centers are directly involved in bond formation and dissociation, even for large molecules. Therefore, we designate these special atoms as ‘active atoms’ and build AC matrices on the basis of these active atoms. Fig. 3a shows an example of active atoms for the hydroformylation reaction, as denoted by red circles. Then, the initial AC matrix of the reactants and corresponding conversion matrices are reduced to the ones on the basis of only the eight active atoms as shown in Fig. 3b. Once the enumeration of AC matrices is completed, their basis transformation from active-atom to all-atom is followed to obtain all-atom AC matrices. Resulting matrices may contain a single molecule or several molecules. In the latter case, they are decomposed into several block matrices, each of which corresponds to a single molecule.

### 2. Step 3: construction of reaction network

#### Construction of reaction network

**Fig. 4:**
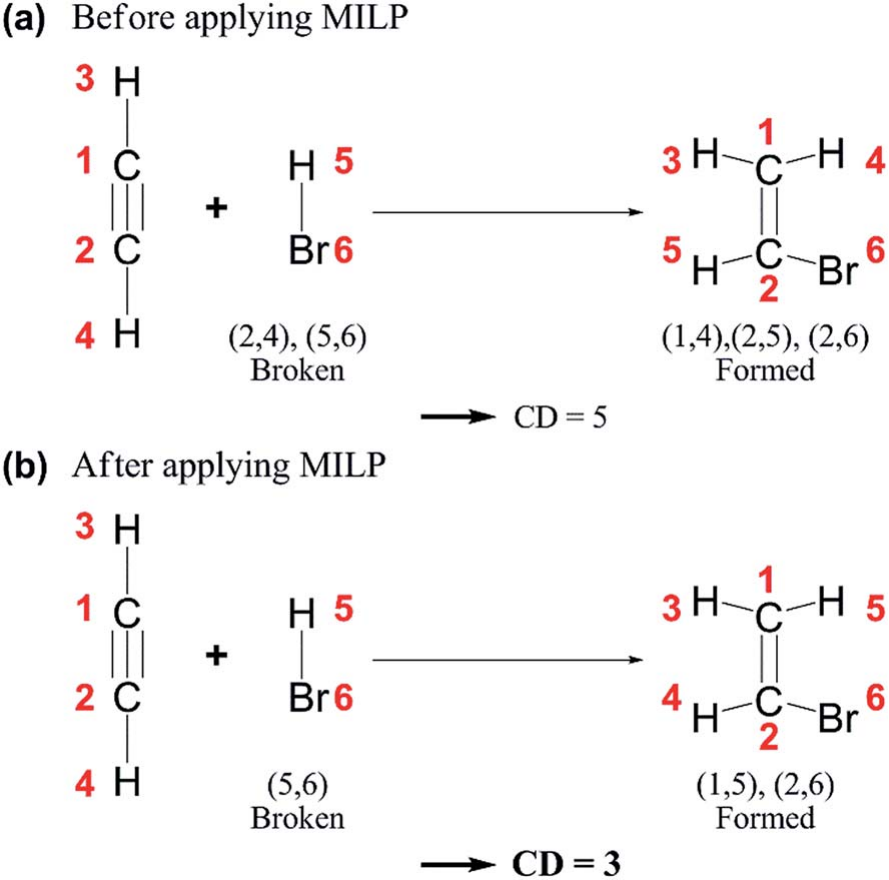
Chemical distances (CDs) of an example reaction (a) before and (b) after applying mixed-integer linear programming (MILP).

A reaction network can be made by connecting remaining intermediates after the structure conversion. Since we are interested in the most favorable reaction path starting from reactants to products, a subnetwork including both the reactants and products is crucial. A small subnetwork may not include important intermediates, whereas a large one entails computational costs. Thus, we need to determine an appropriate range of subnetwork to compromise between accuracy and efficiency prior to constructing the reaction network. We invoke the so-called principle of minimum structure change, which states that most chemical reactions proceed along a pathway with minimum dissociation and formation of bonds.^68^,^69^ This heuristic rule, often regarded as a principle, has been applied to various chemical problems including the elucidation of reaction mechanism.^68^,^69^ To implement this idea in our method, we devised a novel way of discarding intermediates that are placed too far from reactants and products in a reaction network. The concept of chemical distance (CD)^68^,^69^ is used to perform such geometric analysis, which is defined as4
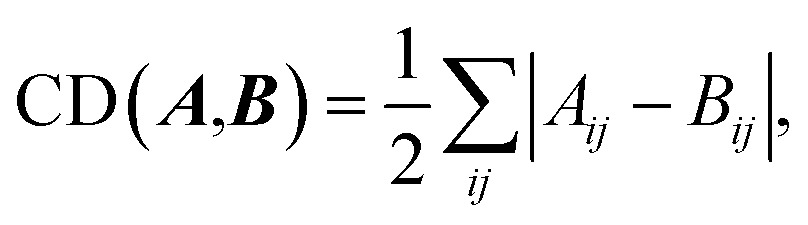
where ***A*** and ***B*** denote the AC matrices of two intermediates, respectively. The CD gives the minimum number of bond changes needed to transform ***A*** into ***B***. It can be overestimated due to the permutation between the two AC matrices, as illustrated in Fig. 4a. This problem can be resolved by calculating the minimum CD out of all possible combinations using the mixed-integer linear programming (MILP) scheme with appropriate variables and objective functions,^70^ as shown in Fig. 4b.

**Fig. 5:**
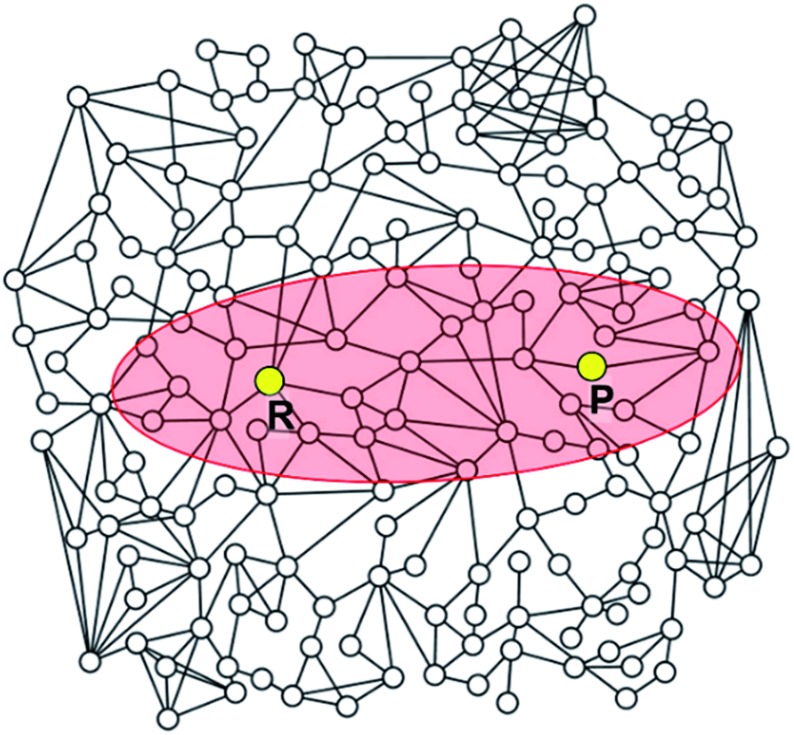
Schematic illustration of a reaction network. The elliptic region shaded in red includes intermediates satisfying the criterion defined by eqn (5). Intermediates located outside the ellipse will not be included in the construction of the reaction network.

Then, we collect all intermediates that satisfy the following criterion:

5 CD(***R***,***I***) + CD(***I***,***P***) ≤ CD(***R***,***P***) + *Δ*,

where *Δ* is called the ‘digression factor’. This criterion determines intermediates that are located inside an ellipse whose focal points correspond to ***R*** and ***P*** in the reaction network, as illustrated in Fig. 5. We note that the screening criterion using eqn (5) can also be applied during the combinatorial generation of AC matrices to further accelerate the process for large systems. The factor *Δ* can be regarded as a convergence parameter which is determined in a way so that top ranked reaction paths in the final stage do not change.

The remaining intermediates are used to build a reaction network. These intermediates are regarded as vertices in the network. It should be noted that molecular conformers having an identical AC matrix share a single vertex. However, they can be treated independently as evaluating the activation energy of each conformer in the next step. The network with *N* intermediates can have *N*(*N* – 1)/2 connections, which makes the process computationally demanding for a large value of *N*. To find kinetically appropriate elementary reactions, the same criteria used in Step 2 for the intermediate sampling are applied. That is, only unimolecular or bimolecular reactions are allowed, and the numbers of bond dissociation/formation should be smaller than or equal to predefined maximum values. In most cases, the maximum value is set to two as in eqn (3), but it is kept as small as possible to reduce computational costs. For catalytic reactions, elementary reactions not involving catalysts are not favored and hence are ignored in the network.

#### Extraction of the minimal reaction network

The distance between two vertices, *i.e.*, the length of an edge, should be related to the activation energy of the corresponding elementary reaction step. The activation energy can be calculated by using conventional quantum chemical methods. However, large computational costs are inevitable to deal with a number of elementary reactions. We note that it would be sufficient to use the CD given by eqn (4) as the distance between vertices for the purpose of first screening. According to the principle of minimum structure change,^68^,^69^ the more the molecular structure changes, the higher the activation energy. Based on this idea, we first obtain the shortest reaction path passing through a specific reaction intermediate including all equidistant ones using the Dijkstra and Yen algorithms.^71^,^72^ The same procedure is repeated for all intermediates in the reaction network, resulting in various reaction paths. Subsequently, all edges not belonging to the sampled paths are disconnected. If the network is decomposed into several subnetworks fully disconnected with each other, only the one containing both reactants and products is regarded as the minimal reaction network that can be determined without using quantum chemical calculations, while all the others are discarded. If the shortest paths are not sufficient, it is straightforward to extend to the second and the third shortest paths. However, this extension does not necessarily increase the network size because they share many vertices and edges with each other.

### 3. Step 4: kinetic analysis of reaction network

The minimal reaction network may have a few tens of reaction paths or more. To find the true minimum energy path out of them, we apply conventional transition state search algorithms to them. At this stage, we are able to take into account all possible molecular conformers or stereoisomers corresponding to a given vertex as described above. Each of them is subjected to the transition state search algorithm using DFT. To minimize computational load, we first consider most frequently appeared edges in the reaction paths. If the activation energy of an edge is above a given threshold, all the reaction paths including the edge are removed from the network. Also, the energy cutoff based on the DFT results can be applied to all intermediates. The remaining ones are considered as the most favorable reaction paths.

All the above procedures were implemented in our code, namely ACE-reaction, using Python 2.7,^73^ with NumPy and SciPy,^74^ and OpenOpt package^75^ as the MILP solver. Our code can be combined with any structure-conversion and electronic structure calculation program. At present, we used the Pybel^76^,^77^ for structure conversion, and DFTB+,^78^ or GAUSSIAN 09 packages^79^ for electronic structure calculations.

## Computational details

We applied our method to two reaction examples: Claisen condensation and cobalt-catalyzed hydroformylation. For the 3D structure conversion of AC matrices sampled in the Claisen reaction, we employed the PM6 semiempirical method^80^ with ethanol solvent described by the CPCM solvation model^81^ as implemented in GAUSSIAN 09.^79^ The density functional tight binding (DFTB) method^78^ was used in the hydroformylation reaction with the trans3d-0-1,^82^ and mio-1-1,^83^ pairwise potential parameters. For DFTB calculations, the maximum numbers of cycles for self-consistent charge (SCC) and geometry relaxation were 500 and 10 000, respectively. The SCC tolerance was set to 10^–5^, and the maximum force value for the geometry optimization was 10^–3^ Hartree Bohr^–1^. The energy profiles of reaction paths for the hydroformylation were further investigated by employing M06 hybrid functional^84^,^85^ with the 6-311++g(d,p) basis set, as implemented in GAUSSIAN 09.^79^

## Results and discussion

### 1. Claisen ester condensation

**Fig. 6:**
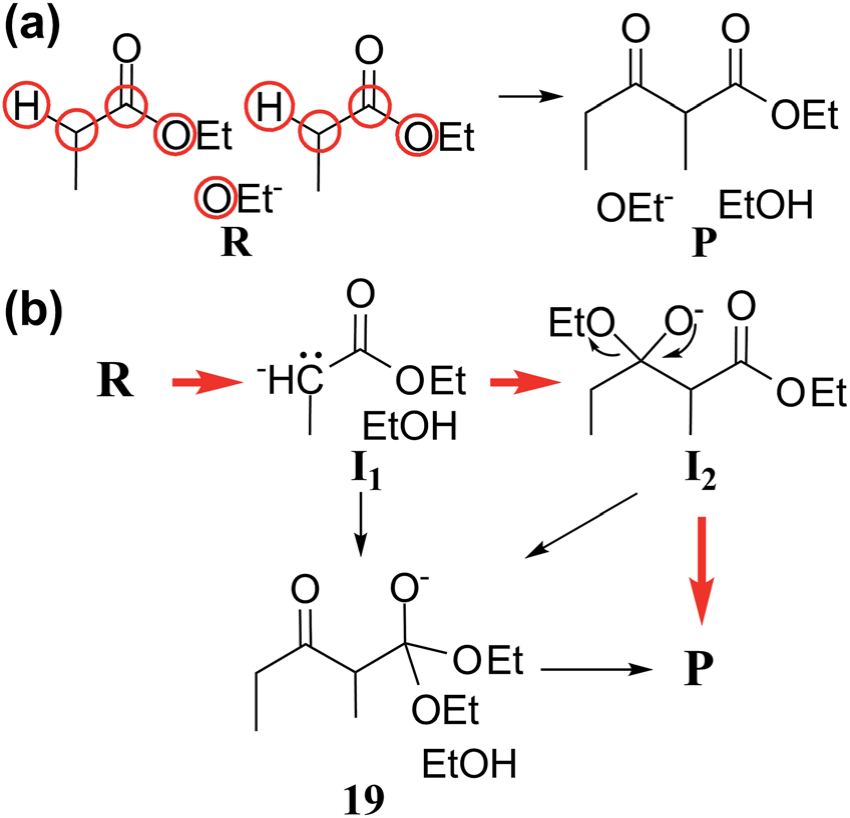
(a) Reactants and products of Claisen ester condensation. The red circles indicate active atoms. (b) Three representative paths predicted by our method. Red arrows indicate the accepted mechanism.

**Fig. 7:**
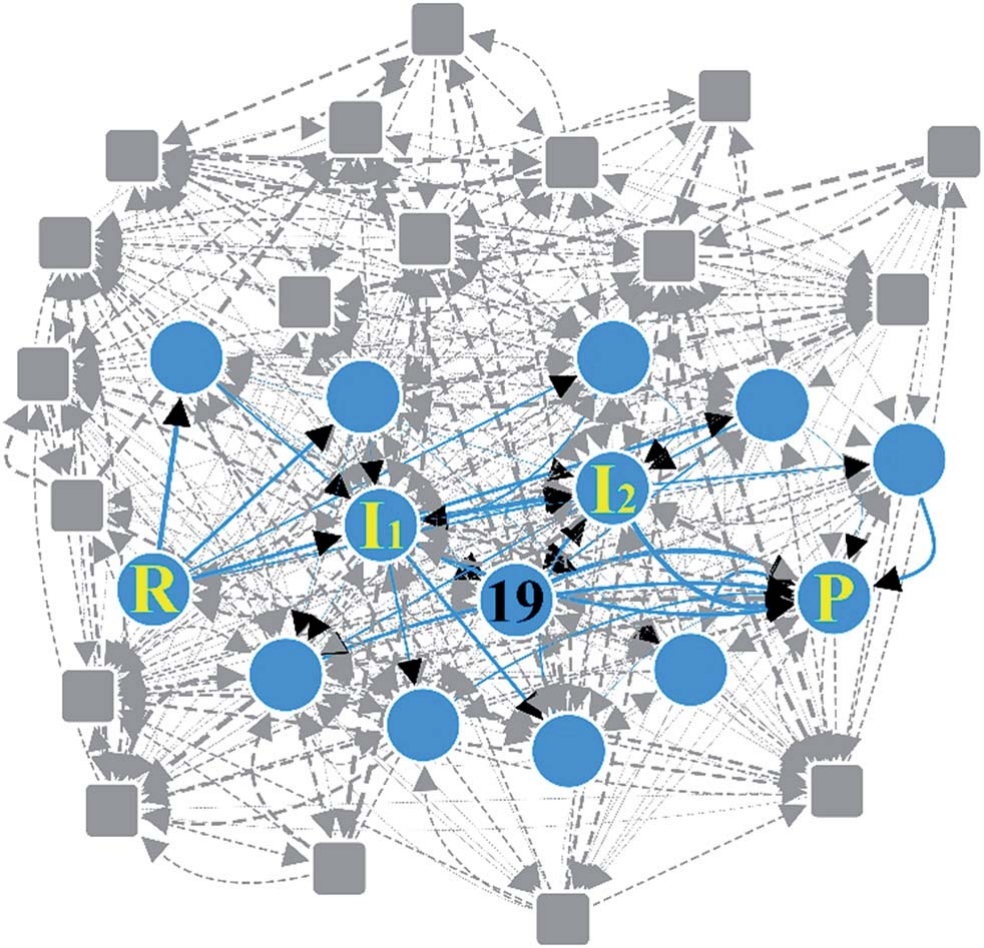
Reaction network of Claisen ester condensation. The circles and solid lines indicate the vertices and edges in the minimal subnetwork obtained in Step 3, respectively. All molecular structures and chemical distance values in the network are available in the ESI.†

Claisen ester condensation is a C–C coupling reaction between two ester molecules in the presence of strong bases. It has been widely utilized in total synthesis and biosynthesis.^86^–^90^ We applied our method to this reaction to test whether or not it is able to find the accepted reaction mechanism. The input parameters and prediction results are summarized in Table 1. In Step 1, we assigned active atoms as shown in Fig. 6a. In Step 2, the combinatorial generation gave 113 intermediates, and 66 were left after screening with the energy tolerance of 20 kcal mol^–1^. In Step 3, they were further screened out by the geometric analysis using eqn (5), resulting in only 32 intermediates. Subsequently, they were connected with each other according to the criterion in eqn (3), leading to a reaction network with 376 edges as shown in Fig. 7. At this stage, the number of intermediates is small enough to handle with quantum chemical methods, but the number of elementary reactions is relatively too large to perform accurate transition state calculations. Thus, we need to further rule out less favorable elementary reactions. We extracted the minimal reaction network composed of the paths within the top 50% in terms of CD using the Dijkstra and Yen algorithms.^71^,^72^ As a result, 29 paths were obtained only with 14 vertices and 35 edges (circles and solid lines in Fig. 7).

**Table 1:** Input parameters and prediction results

Reaction	Step 2		Step 3		Calculation time^*c*^
	*E*_tol_ (kcal mol^–1^)	No. intermediates^*a*^	*Δ*	(*N*_V_, *N*_E_)^*b*^	
Claisen ester condensation	20.0	113 → 66	6	(32, 376) → (14, 35)	55 m 3 s (53 m 57 s + 1 m 6 s)^*d*^
Cobalt-catalyzed hydroformylation	20.0	239 → 224	6	(54, 403) → (39, 104)	56 m 2 s (53 m 35 s + 2 m 27 s)^*d*^

^*a*^Before and after screening with *E*_tol_.

^*b*^Number of vertices (*N*_V_) and edges (*N*_E_) after extraction of minimal reaction network.

^*c*^Intel(R) Xeon(R) CPU E5-2690 v2@2.90 GHz (16 cores).

^*d*^Time taken in Step 2 + Step 3.

Fig. 6b shows three representative paths; the other 26 paths are given in the ESI.† We note that the generally accepted mechanism in organic chemistry (***R*** → ***I***_1_ → ***I***_2_ → ***P***) was included in the minimal reaction network. Surprisingly, this path also corresponds to the first shortest path in terms of CD. The overall process took about 55 minutes on a single workstation (Table 1).

### 2. Cobalt-catalyzed hydroformylation

**Fig. 8:**
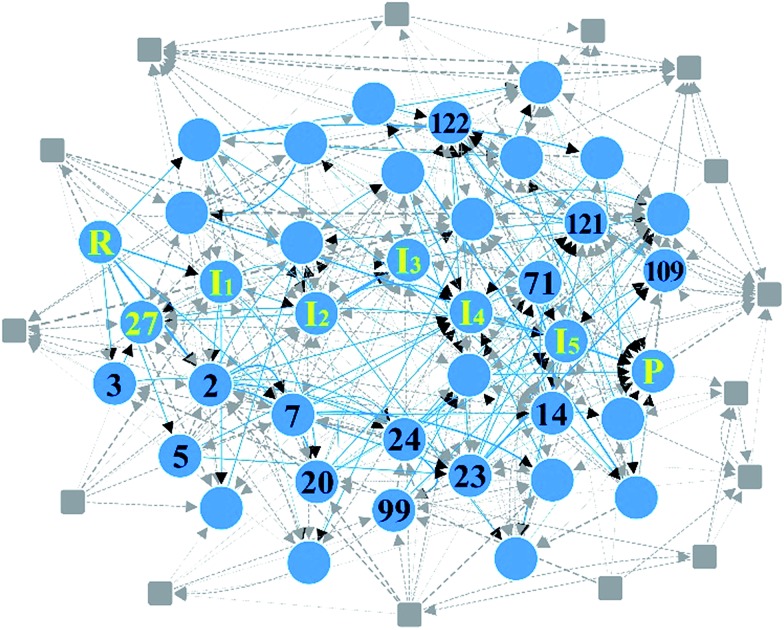
Reaction network of cobalt-catalyzed hydroformylation. The circles and solid lines indicate the vertices and edges in the minimal subnetwork obtained in Step 3, respectively. All molecular structures and chemical distance values in the network are available in the ESI.†

As the second example, we chose the HCo(CO)_3_-catalyzed hydroformylation whose mechanism has been proposed by Heck and Breslow,^91^ because it is a relatively simple organometallic reaction and thus has been widely studied by other automated prediction methods.^10^,^26^,^52^,^53^ In Step 1, we assigned active atoms as illustrated in Fig. 3a. Table 1 summarizes the input parameters and prediction results. Unlike the first example, we had 224 intermediates in Step 2. Here, screening by the energy cutoff was so ineffective that only 15 intermediates have been ruled out. In Step 3, however, filtering with eqn (5) drastically reduced the number of intermediates, leading to a reaction network with 54 vertices and 403 edges as shown in Fig. 8. This indicates that the geometric analysis illustrated in Fig. 5 is practically essential to extract a reaction network with a tractable size from complicated reactions. Then, we further reduced the size of the network so as to contain only the top 50% paths in terms of CD using the Dijkstra algorithm.^71^ The resulting network was composed of 91 paths including 39 vertices and 104 edges (circles and solid lines in Fig. 8). The original Heck–Breslow mechanism^91^ appeared within the top 33%. The other paths in the top 50% are given in the ESI.† It should be noted that we were able to arrive at this small network without quantum calculations at the first principle level, so that the whole procedure took only around 56 minutes (Table 1) on a single workstation. Most of the time was used for the on-the-fly 3D geometry optimization of all intermediates using DFTB in Step 2. We note that the intermediates in the blue circle of Fig. 8 were also discovered by the automated prediction method in ref. 53. However, it obtained those after 45 simulations where each simulation took about 12 hours in the molecular dynamics sampling of reaction paths.

**Fig. 9:**
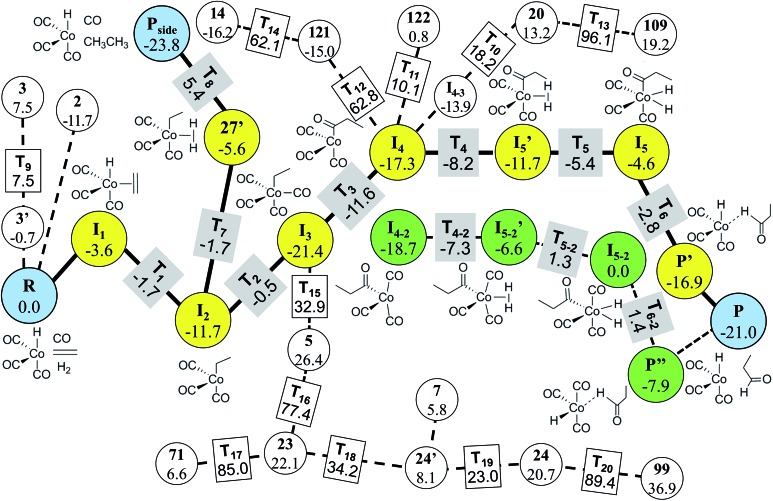
Final reaction network for the hydroformylation reaction obtained at the DFT level. The circles indicate the reactants, products, and intermediates, while the rectangles indicate the transition states. The thick lines and the yellow circles denote the Heck–Breslow mechanism to give the product (***P***) and the hydrogenation mechanism to form the side product (***P***_**side**_). The other paths are drawn with dashed lines. The green circles indicate the stereoisomers of the intermediates in the Heck–Breslow mechanism. The numbers in the circles and the rectangles denote the relative energies of intermediates and transition states with respect to that of the reactant (***R***), respectively. All the energy values are in kcal mol^–1^.

In Step 4, we performed DFT calculations for the 39 vertices in the minimal reaction network. Based on the DFT results, we removed vertices and edges using the following two criteria before transition state calculations: energy tolerance (*E*_tol_) of 20 kcal mol^–1^ for intermediates and endothermic reactions with energy difference of 20 kcal mol^–1^ for vertices (those values can be changed according to reaction conditions). Isolated vertices in the network after applying the two criteria were also discarded. As a result, only 29 vertices and 74 edges were left. They were subjected to transition state calculations using DFT at the experimental temperature (403.15 K) and pressure (200 atm). At this step, we considered all possible conformers for each intermediate. Fig. 9 displays the final reaction network obtained from the DFT study. The numbers in the circles and the rectangles denote the relative energies of intermediates and transition states with respect to that of the reactants, respectively. The Cartesian coordinates of all the molecules and transition states optimized at the DFT level are available in the ESI.† Indeed, the Heck–Breslow mechanism (yellow circles connecting ***R*** and ***P*** in Fig. 9) was turned out to be kinetically the most favorable; the activation energy of the rate-determining step ***I***_2_ → ***I***_3_ is 11.2 kcal mol^–1^. We were able to find a path involving conformers or stereoisomers such as ***I***_4–2_ and ***I***_5–2_ (the green circles in Fig. 9), which was also reported in ref. 53. However, this path was not directly reachable from the reactants. In addition, the hydrogenation path as a well-known side reaction was also included there (the yellow circles connecting ***R*** and ***P***_**side**_ in Fig. 9).^26^,^92^ ***P***_**side**_ was also sampled by our method (vertex **1**, see the ESI†), but was screened in Step 3. Nonetheless, it was readily derived from the intermediate **27′** in DFT calculations. The path linking to the intermediate **14** is also related to the hydrogenation mechanism, but it is kinetically unfavorable due to the very high barrier of over 60 kcal mol^–1^.

It is emphasized that our method is much more efficient than the previous approaches. For the same hydroformylation reaction, we performed only 36 transition state searches including failed ones at the DFT level, whereas Maeda’s approach performed 2266 Hessian calculations^10^ and Varela’s method dealt with 448 elementary reactions.^26^ However, our approach has some limitations; intermediates such as ***I***_5_′ and **27′** obtained from the DFT calculations did not appear in the combinatorial generation of intermediates because they have unusual chemical bonds such as H with two single bonds. It is possible to sample them by modifying the screening criteria, but then it will produce a lot of undesired molecules. Consequently, final reaction paths predicted from the graph-theoretic method should be refined through accurate quantum calculations.

## Conclusions

We developed an efficient graph-theoretic method for the automated prediction of reaction mechanism. It is based on the fact that chemical reactions can be described by the successive changes in chemical bonds. Reactant molecules can be represented with atom connectivity (AC) matrices. Then, hypothetical intermediates can be sampled through the combinatorial enumeration of the matrix. Among them, chemically inappropriate AC matrices are discarded by on-the-fly 3D structure conversion and pruning criteria. In addition, the geometric analysis based on a chemical distance concept is used to further screen out intermediates whose structures are substantially different from reactants and products. The remaining molecules are regarded as vertices and connected to build a reaction network. The key feature of our method is to extract a minimal subnetwork from a very complex full network. To this end, we explore the reaction pathways connecting reactants and products with minimum dissociation and formation of chemical bonds for all intermediates in the network using the Dijkstra algorithm. Subsequently, they are subjected to accurate transition state calculations for refinements. It should be emphasized that though our method relies on chemical heuristics, the rules imposed for enumeration and screening are not reaction specific. Therefore, it can be applied to a wide range of chemical reactions.

The efficiency and reliability of our method have been assessed by applying it to two example reactions. It was able to successfully predict the accepted reaction mechanism of Claisen ester condensation. Also, it could find not only the original Heck–Breslow mechanism, but also the hydrogenation of ethylene as a side reaction for cobalt-catalyzed hydroformylation, showing its potential applicability to organometallic or inorganic reactions. It is remarkable that for both examples, our method completed the whole process, except for DFT calculations, within only an hour on a single workstation with 16 Intel Xeon cores.

The present work offers an efficient approach to predict reaction pathways. However, the following issues need to be addressed to further improve its reliability and applicability. First of all, molecular graphs have clear limitations to discriminate different electronic states of molecules with an identical AC. For instance, one can recall pre-reaction and post-reaction complexes, ion pairs, charge-transfer complexes, and many other types of spatial configurations of nuclei, which correspond to deep local minima in potential energy surfaces. Second, more rigorous catalytic effects need to be included throughout the process from combinatorial generation to distance evaluation. Chemical bonds between organic substrates and metal catalysts are often ill-defined. As a result, it is difficult to apply simple chemical rules such as atomic valences and formal charges to the combinatorial enumeration step. As future works, we expect that a novel combination of heuristic rules, first principles theory, and machine learning techniques will be a key to resolve the aforementioned problems.

## Conflicts of interest

The authors declare no competing financial interest.

## Supplementary Material

Supplementary information
