# Supplementary material for: Efficient prediction of reaction paths through molecular graph and reaction network analysis
Source: Chem Sci. 2017 Dec 12;9(4):825–35. doi: 10.1039/c7sc03628k (PMC5887236; doi:10.1039/c7sc03628k)
Supplement: Supplementary file 1 [file SC-009-C7SC03628K-s001.pdf]

Electronic Supplementary Material (ESI) for Chemical Science.
This journal is © The Royal Society of Chemistry 2017

[Electronic Supplementary Information]

Efficient prediction of reaction paths through molecular graph and reaction network analysis

Yeonjoon Kim, Jin Woo Kim, Zeehyo Kim, and Woo Youn Kim*

Department of Chemistry, KAIST, 291 Daehak-ro, Yuseong-gu, Daejeon 34141, Korea

*E-mail: wooyoun@kaist.ac.kr (WYK)

1. Detailed information on reaction networks and pathways for two example reactions

1.1. Claisen ester condensation.

Table S1. Information on the vertices of the reaction network obtained in Step 3 for the Claisen ester condensation.

Vertex name	SMILES	Included in the extracted minimal network?
R	[C]([CH2][CH3])([O][CH2][CH3])=[O].[C]([CH2][CH3])([O][CH2][CH3])=[O].[O-][CH2][CH3]	Yes
P	[C]([CH2][CH3])([CH]([CH3])[C]([O][CH2][CH3])=[O])=[O].[O-][CH2][CH3].[OH][CH2][CH3]	Yes
I_1	[C]([CH-][CH3])([O][CH2][CH3])=[O].[C]([CH2][CH3])([O][CH2][CH3])=[O].[OH][CH2][CH3]	Yes
I_2	[C]([CH]([C]([CH2][CH3])([O][CH2][CH3])[O-])[CH3])([O][CH2][CH3])=[O].[OH][CH2][CH3]	Yes
1	[C]([CH2][CH3])([O][CH2][CH3])=[O].[C]([CH2][CH3])([O][CH2][CH3])([O][CH2][CH3])[O-]	Yes
2	[C-]([O][CH2][CH3])=[O].[CH2]([O][CH2][CH3])[CH3].[C]([CH2][CH3])([O][CH2][CH3])=[O]	Yes
5	[CH]([CH]([O][CH2][CH3])[CH3])([O][CH2][CH3])[O-].[C]([CH2][CH3])([O][CH2][CH3])=[O]	Yes
16	[C]([CH2][CH3])([O][CH2][CH3])([O][CH2][CH3])[O-].[C](=[CH][CH3])=[O].[OH][CH2][CH3]	Yes
19	[C]([CH2][CH3])([CH]([CH3])[C]([O][CH2][CH3])([O][CH2][CH3])[O-])=[O].[OH][CH2][CH3]	Yes
26	[C-]([CH]([CH3])[C]([O][CH2][CH3])=[O])=[O].[CH2]([O][CH2][CH3])[CH3].[OH][CH2][CH3]	Yes
30	[C-]([O][CH2][CH3])=[O].[CH]([C]([CH2][CH3])=[O])([O][CH2][CH3])[CH3].[OH][CH2][CH3]	Yes
50	[C]([CH]([CH2][CH3])[CH3])([O][CH2][CH3])=[O].[OH][CH2][CH3].[C-]([O][CH2][CH3])=[O]	Yes
51	[C]([CH]([O][CH2][CH3])[CH3])([O][CH2][CH3])([C]([CH2][CH3])=[O])[O-].[OH][CH2][CH3]	Yes
56	[C]([CH-][CH3])([CH]([CH3])[C]([O][CH2][CH3])=[O])=[O].[OH][CH2][CH3].[OH][CH2][CH3]	Yes
4	[C]([O][CH2][CH3])([C]([CH2][CH3])([O][CH2][CH3])[O-])=[O].[CH2]([O][CH2][CH3])[CH3]	No
10	[CH]([O][CH2][CH3])=[O].[CH2]([CH]([CH3])[C]([O][CH2][CH3])=[O])[CH3].[O-][CH2][CH3]	No
13	[CH]([CH]([O][CH2][CH3])[CH3])=[O].[C]([CH2][CH3])([O][CH2][CH3])=[O].[O-][CH2][CH3]	No
22	[CH]([C]([CH2][CH3])([O][CH2][CH3])[O-])([O][CH2][CH3])[CH3].[CH]([O][CH2][CH3])=[O]	No
23	[CH]([O][CH2][CH3])=[O].[CH2]([O][CH2][CH3])[CH3].[C]([CH-][CH3])([O][CH2][CH3])=[O]	No
24	[C]([O][CH2][CH3])([CH]([CH3])[CH]([O][CH2][CH3])[O-])=[O].[CH2]([O][CH2][CH3])[CH3]	No
32	[C]([O][CH2][CH3])([C](=[CH][CH3])[O-])=[O].[CH2]([O][CH2][CH3])[	No

	CH3].[OH][CH2][CH3]	
36	[C]([O][CH2][CH3])([C]([CH]([CH3])[CH2][CH3])([O][CH2][CH3])[O-])=[O].[OH][CH2][CH3]	No
40	[C]([O][CH2][CH3])([C]([CH]([CH3])[CH2][CH3])=[O])=[O].[O-][CH2][CH3].[OH][CH2][CH3]	No
46	[C]([O][CH2][CH3])([C]([O-])([CH2][CH3])[CH]([O][CH2][CH3])[CH3])=[O].[OH][CH2][CH3]	No
49	[C]([CH]([O][CH2][CH3])[CH3])([C]([CH2][CH3])([O][CH2][CH3])[O-])=[O].[OH][CH2][CH3]	No
52	[C]([CH]([CH]([CH3])[CH]([O][CH2][CH3])[O-])[CH3])([O][CH2][CH3])=[O].[OH][CH2][CH3]	No
55	[C](=[CH][CH3])=[O].[O]([CH]([CH3])[CH]([O][CH2][CH3])[O-])[CH2][CH3].[OH][CH2][CH3]	No
57	[CH]([CH]([CH]([CH3])[C]([O][CH2][CH3])=[O])[CH3])=[O].[O-][CH2][CH3].[OH][CH2][CH3]	No
58	[CH]([CH]([O][CH2][CH3])[CH3])=[O].[C]([CH-][CH3])([O][CH2][CH3])=[O].[OH][CH2][CH3]	No
60	[C]([CH]([CH]([CH]([O][CH2][CH3])[CH3])[O-])[CH3])([O][CH2][CH3])=[O].[OH][CH2][CH3]	No
61	[CH]([CH]([C]([CH]([O][CH2][CH3])[CH3])=[O])[CH3])([O][CH2][CH3])[O-].[OH][CH2][CH3]	No
62	[CH]([O][CH2][CH3])=[O].[CH]([C]([CH2][CH3])=[O])([O][CH2][CH3])[CH3].[O-][CH2][CH3]	No

Table S2. Information on the edges of the reaction network obtained in Step 3 for the Claisen ester condensation.

Source	Target	Chemical distance	Included in the extracted minimal network?
R	1	1	Yes
R	2	2	Yes
R	I_1	2	Yes
R	I_2	3	Yes
R	5	3	Yes
I_1	P	2	Yes
I_2	P	1	Yes
16	P	4	Yes
19	P	1	Yes
26	P	2	Yes
30	P	2	Yes
50	P	4	Yes
51	P	3	Yes
56	P	2	Yes
1	I_1	3	Yes
1	I_2	4	Yes
1	19	4	Yes
2	I_1	4	Yes

2	26	4	Yes
2	30	4	Yes
I_1	I_2	1	Yes
5	I_1	3	Yes
I_1	16	2	Yes
I_1	19	3	Yes
I_1	30	4	Yes
I_1	50	2	Yes
I_1	51	3	Yes
I_1	56	4	Yes
5	I_2	4	Yes
16	I_2	3	Yes
I_2	19	2	Yes
I_2	30	3	Yes
50	I_2	3	Yes
I_2	56	3	Yes
16	19	3	Yes
2	R	2	No
R	4	3	No
4	R	3	No
I_1	R	2	No
I_2	R	3	No
5	R	3	No
P	I_1	2	No
P	I_2	1	No
P	16	4	No
P	19	1	No
P	26	2	No
P	30	2	No
P	32	4	No
32	P	4	No
P	46	3	No
46	P	3	No
P	50	4	No
P	51	3	No
P	55	4	No
55	P	4	No
P	56	2	No
P	58	4	No
58	P	4	No
P	60	3	No
60	P	3	No
P	61	3	No
61	P	3	No

62	61	3	No
2	1	3	No
1	4	4	No
4	1	4	No
I_1	1	3	No
I_2	1	4	No
1	5	4	No
5	1	4	No
16	1	3	No
19	1	4	No
60	61	2	No
61	60	2	No
2	4	1	No
4	2	1	No
I_1	2	4	No
2	5	3	No
5	2	3	No
2	13	4	No
13	2	4	No
2	22	3	No
22	2	3	No
2	23	2	No
23	2	2	No
2	24	3	No
24	2	3	No
26	2	4	No
30	2	4	No
2	32	4	No
32	2	4	No
2	50	4	No
50	2	4	No
58	60	1	No
60	58	1	No
58	61	3	No
61	58	3	No
4	5	4	No
5	4	4	No
4	22	4	No
22	4	4	No
4	23	3	No
23	4	3	No
4	24	4	No
24	4	4	No
4	32	3	No
32	4	3	No
4	36	4	No

36	4	4	No
4	46	4	No
46	4	4	No
4	49	4	No
49	4	4	No
4	51	4	No
51	4	4	No
57	58	2	No
58	57	2	No
57	60	3	No
60	57	3	No
I_2	I_1	1	No
I_1	5	3	No
13	I_1	4	No
16	I_1	2	No
19	I_1	3	No
I_1	23	4	No
23	I_1	4	No
26	I_1	4	No
30	I_1	4	No
I_1	32	4	No
32	I_1	4	No
I_1	36	3	No
36	I_1	3	No
40	I_1	4	No
I_1	49	3	No
49	I_1	3	No
50	I_1	2	No
51	I_1	3	No
I_1	52	3	No
52	I_1	3	No
55	I_1	4	No
56	I_1	4	No
57	I_1	4	No
I_1	58	4	No
58	I_1	4	No
57	56	4	No
56	58	4	No
58	56	4	No
56	60	3	No
60	56	3	No
56	61	3	No
61	56	3	No
I_2	5	4	No
19	I_2	2	No
I_2	22	4	No

22	I_2	4	No
I_2	24	4	No
24	I_2	4	No
26	I_2	3	No
30	I_2	3	No
I_2	36	4	No
36	I_2	4	No
I_2	46	4	No
46	I_2	4	No
I_2	49	4	No
49	I_2	4	No
I_2	50	3	No
I_2	51	4	No
51	I_2	4	No
I_2	52	4	No
52	I_2	4	No
56	I_2	3	No
I_2	60	4	No
60	I_2	4	No
I_2	61	4	No
61	I_2	4	No
55	56	4	No
55	57	4	No
57	55	4	No
55	58	2	No
58	55	2	No
55	60	3	No
55	61	3	No
13	5	1	No
5	22	2	No
22	5	2	No
5	23	3	No
23	5	3	No
5	24	4	No
24	5	4	No
5	49	4	No
49	5	4	No
5	51	4	No
51	5	4	No
5	52	4	No
52	5	4	No
55	5	3	No
5	58	3	No
58	5	3	No
5	60	4	No
60	5	4	No

62	5	3	No
55	52	3	No
57	52	1	No
58	52	3	No
52	60	4	No
60	52	4	No
52	61	4	No
61	52	4	No
10	22	3	No
22	10	3	No
10	23	2	No
23	10	2	No
10	24	3	No
24	10	3	No
10	36	3	No
10	50	2	No
50	10	2	No
10	52	3	No
51	55	3	No
55	51	3	No
13	22	3	No
22	13	3	No
13	49	3	No
13	58	2	No
58	13	2	No
13	60	3	No
50	52	3	No
52	50	3	No
57	50	4	No
19	16	3	No
16	26	4	No
26	16	4	No
16	30	4	No
30	16	4	No
16	32	4	No
32	16	4	No
16	40	4	No
40	16	4	No
16	49	3	No
49	16	3	No
16	50	4	No
50	16	4	No
16	55	4	No
55	16	4	No
16	56	4	No
56	16	4	No

16	57	4	No
57	16	4	No
16	58	4	No
58	16	4	No
49	51	2	No
51	49	2	No
49	58	3	No
58	49	3	No
49	60	4	No
60	49	4	No
49	61	4	No
61	49	4	No
26	19	3	No
19	30	3	No
30	19	3	No
19	46	4	No
46	19	4	No
19	51	4	No
51	19	4	No
19	56	3	No
56	19	3	No
19	60	4	No
60	19	4	No
19	61	4	No
61	19	4	No
46	49	2	No
49	46	2	No
46	51	2	No
51	46	2	No
46	60	4	No
60	46	4	No
46	61	4	No
61	46	4	No
46	62	3	No
62	46	3	No
22	23	3	No
23	22	3	No
22	24	4	No
24	22	4	No
22	30	3	No
30	22	3	No
22	46	4	No
46	22	4	No
22	61	4	No
61	22	4	No
22	62	1	No

62	22	1	No
40	46	3	No
46	40	3	No
40	49	3	No
49	40	3	No
40	50	2	No
23	24	1	No
24	23	1	No
23	26	4	No
26	23	4	No
23	32	4	No
32	23	4	No
23	58	4	No
58	23	4	No
23	62	4	No
62	23	4	No
40	36	1	No
36	46	4	No
46	36	4	No
36	49	4	No
49	36	4	No
36	50	1	No
50	36	1	No
36	51	4	No
51	36	4	No
36	52	4	No
52	36	4	No
26	24	3	No
24	60	4	No
60	24	4	No
24	61	4	No
61	24	4	No
32	36	3	No
36	32	3	No
32	40	2	No
40	32	2	No
32	46	3	No
46	32	3	No
32	49	3	No
49	32	3	No
32	50	4	No
50	32	4	No
32	51	3	No
51	32	3	No
32	55	4	No
55	32	4	No

26	30	4	No
30	26	4	No
26	32	2	No
32	26	2	No
26	40	4	No
40	26	4	No
26	50	4	No
26	55	4	No
55	26	4	No
26	56	4	No
26	58	4	No
58	26	4	No
26	60	3	No
26	61	3	No
30	32	4	No
32	30	4	No
30	40	4	No
40	30	4	No
30	46	1	No
46	30	1	No
30	49	3	No
49	30	3	No
30	50	4	No
50	30	4	No
30	51	3	No
51	30	3	No
30	55	4	No
55	30	4	No
30	56	4	No
56	30	4	No
30	58	4	No
58	30	4	No
30	60	3	No
60	30	3	No
30	61	3	No
61	30	3	No
30	62	2	No
62	30	2	No

Table S3. The 29 predicted reaction paths of the Claisen ester condensation.

Paths	Sum of chemical distances
$R \rightarrow I_1 \rightarrow I_2 \rightarrow P$ (Accepted mechanism)	4
$R \rightarrow I_1 \rightarrow P$	4
$R \rightarrow I_2 \rightarrow P$	4
$R \rightarrow 1 \rightarrow I_1 \rightarrow P$	6

R→1→19→P	6
R→1→I_1→I_2→P	6
R→1→I_2→P	6
R→I_1→19→P	6
R→I_2→19→P	6
R→I_1→I_2→19→P	6
R→2→30→P	8
R→2→26→P	8
R→2→I_1→P	8
R→2→I_1→I_2→P	8
R→5→I_1→P	8
R→5→I_2→P	8
R→5→I_1→I_2→P	8
R→I_1→16→P	8
R→I_1→16→19→P	8
R→I_1→16→I_2→P	8
R→I_1→30→P	8
R→I_1→I_2→30→P	8
R→I_2→30→P	8
R→I_1→50→P	8
R→I_1→50→I_2→P	8
R→I_1→51→P	8
R→I_1→56→P	8
R→I_2→56→P	8
R→I_1→I_2→56→P	8

1.2. Cobalt-catalyzed hydroformylation.

Table S4. Information on the vertices of the reaction network obtained in Step 3 for the cobalt-catalyzed hydroformylation.

Vertex name	SMILES	Included in the extracted minimal network?
R	[CoH](=[C]=[O])(=[C]=[O])=[C]=[O].[CH2]=[CH2].[C]#[O].[HH]	Yes
P	[CoH](=[C]=[O])(=[C]=[O])=[C]=[O].[CH2]([CH]=[O])[CH3]	Yes
I_1	[CoH](=[C]=[O])(=[C]=[O])([CH2]1)([CH2]1)=[C]=[O].[C]#[O].[HH]	Yes
I_2	[Co](=[C]=[O])(=[C]=[O])([CH2][CH3])=[C]=[O] .[C]#[O].[HH]	Yes
I_3	[Co](=[C]=[O])(=[C]=[O])([CH2][CH3])(=[C]=[O])=[C]=[O].[HH]	Yes
I_4	[Co](=[C]=[O])(=[C]=[O])([C](=[O])[CH2][CH3])=[C]=[O].[HH]	Yes
I_5	[CoH2](=[C]=[O])(=[C]=[O])([C](=[O])[CH2][CH3])=[C]=[O]	Yes
2	[CoH](=[C]=[O])(=[C]=[O])(=[C]=[O])=[C]=[O].[CH2]=[CH2].[HH]	Yes
3	[CoH3](=[C]=[O])(=[C]=[O])=[C]=[O].[CH2]=[CH2].[C]#[O]	Yes
5	[Co](=[C]=[O])(=[C]=[O])(#[C][OH])=[C]=[O].[CH2]=[CH2].[HH]	Yes

7	[Co](=[C]=[O])(=[C]=[O])([CH]=[O])=[C]=[O].[CH2]=[CH2].[HH]	Yes
8	[CoH](=[C]=[O])(=[C]=[O])(=[CH2])=[C]=[O].[CH2]=[C]=[O].[HH]	Yes
12	[CoH](=[C]=[O])(=[C]=[O])(=[C]=[O])=[C]=[O].[CH3][CH3]	Yes
14	[Co](=[C]=[O])(=[C]=[O])([CH]=[O])=[C]=[O].[CH3][CH3]	Yes
20	[CoH]1(=[C]=[O])(=[C]=[O])([CH2][CH2][C]1=[O])=[C]=[O].[HH]	Yes
22	[Co](=[C]=[O])(=[C]=[O])(#[C][O][CH2][CH3])=[C]=[O].[HH]	Yes
23	[CoH](=[C]=[O])(=[C]=[O])(=[CH][OH])=[C]=[O].[CH2]=[CH2]	Yes
24	[CoH2](=[C]=[O])(=[C]=[O])([CH]=[O])=[C]=[O].[CH2]=[CH2]	Yes
27	[CoH2](=[C]=[O])(=[C]=[O])([CH2][CH3])=[C]=[O].[C]#[O]	Yes
34	[CoH](=[C]=[O])(=[C]=[O])=[C]=[O].[CH2]([CH]1[OH])[CH2]1	Yes
36	[CoH](=[C]=[O])(=[C]=[O])=[C]=[O].[CH2]=[C]([OH])[CH3]	Yes
40	[CoH](=[C]=[O])(=[C]=[O])([CH2]1)([CH2]1)=[C]=[O].[CH2]=[O]	Yes
49	[Co](=[C]=[O])(=[C]=[O])([CH]([O]1)[CH2][CH2]1)=[C]=[O].[HH]	Yes
54	[CoH]([C]=[O])([C]=[O])([CH2][CH3])=[C]=[O].[C][OH]	Yes
55	[Co](=[C]=[O])(=[C]=[O])([CH2][CH3])=[C]=[O].[CH2]=[O]	Yes
56	[CoH]([C]=[O])([C]=[O])([CH2][CH3])=[C]=[O].[CH]=[O]	Yes
57	[CoH](=[C]=[O])(=[C]=[O])(=[CH2])=[C]=[O].[CH4].[C]#[O]	Yes
60	[Co](=[C]=[O])(=[C]=[O])([CH3])=[C]=[O].[CH2]=[C]=[O].[HH]	Yes
64	[CoH](=[C]=[O])(=[C]=[O])(=[CH2])=[C]=[O].[CH2]=[CH][OH]	Yes
65	[CoH]([C]=[O])([C]=[O])([CH3])=[C]=[O].[CH2]=[C][OH]	Yes
66	[CoH](=[C]=[O])(=[C]=[O])(=[CH2])=[C]=[O].[CH3][CH]=[O]	Yes
67	[CoH2](=[C]=[O])(=[C]=[O])([CH3])=[C]=[O].[CH2]=[C]=[O]	Yes
71	[CoH](=[C]=[O])(=[C]=[O])([CH2]1)([O]1)=[C]=[O].[CH2]=[CH2]	Yes
99	[CoH2](=[C]=[O])(=[C]=[O])([CH]([O]1)[CH2][CH2]1)=[C]=[O]	Yes
109	[CoH](=[C]=[O])(=[C]=[O])([CH2][CH2]1)([CH]1[OH])=[C]=[O]	Yes
117	[CoH](=[C]=[O])(=[C]=[O])(=[CH][O][CH2][CH3])=[C]=[O]	Yes
121	[Co](=[C]=[O])(=[C]=[O])([CH]([OH])[CH2][CH3])=[C]=[O]	Yes
122	[CoH](=[C]=[O])(=[C]=[O])(=[C]([OH])[CH2][CH3])=[C]=[O]	Yes
128	[CoH](=[C]=[O])(=[C]=[O])([CH2][CH3])([CH]=[O])=[C]=[O]	Yes
1	[CoH](=[C]=[O])(=[C]=[O])=[C]=[O].[CH3][CH3].[C]#[O]	No
10	[CoH](=[C]=[O])(=[C]=[O])=[C]=[O].[CH2]=[CH2].[CH2]=[O]	No
15	[CoH](=[C]=[O])(=[C]=[O])=[C]=[O].[CH3][C](=[O])[CH3]	No
19	[CoH](=[C]=[O])(=[C]=[O])(=[C]([O]1)[CH2][CH2]1)=[C]=[O].[HH]	No
26	[CoH3](=[C]=[O])(=[C]=[O])=[C]=[O].[CH2]1[C](=[O])[CH2]1	No
28	[CoH](=[C]=[O])(=[C]=[O])([C]1([CH2]2)[CH2]2)([O]1)=[C]=[O].[HH]	No
30	[Co](=[C]=[O])(=[C]=[O])([O][CH]([CH2]1)[CH2]1)=[C]=[O].[HH]	No
38	[Co]([C]=[O])([C]=[O])([CH2]1)([CH2]1)=[C]=[O].[CH]=[O].[HH]	No
70	[CoH](=[C]=[O])(=[C]=[O])=[C]=[O].[CH2]([CH2]1)[CH2][O]1	No
73	[CoH](=[C]=[O])(=[C]=[O])([CH]1[CH2][CH3])([O]1)=[C]=[O]	No
74	[CoH](=[C]=[O])(=[C]=[O])=[C]=[O].[CH2]=[CH][O][CH3]	No
75	[CoH](=[C]=[O])(=[C]=[O])=[C]=[O].[CH2]([O]1)[CH]1[CH3]	No
76	[CoH](=[C]=[O])(=[C]=[O])([CH3])([O][CH]=[CH2])=[C]=[O]	No
77	[CoH](=[C]=[O])(=[C]=[O])([CH2][CH]=[O])([CH3])=[C]=[O]	No

136	[CoH2](=[C]=[O])(=[C]=[O])([O][CH]([CH2]1)[CH2]1)=[C]=[O]	No

Table S5. Information on the edges of the reaction network obtained in Step 3 for the cobalt-catalyzed hydroformylation.

Source	Target	Chemical distance	Included in the extracted minimal network?
R	2	1	Yes
R	3	3	Yes
R	I_1	2	Yes
R	5	3	Yes
R	7	3	Yes
R	I_2	3	Yes
R	8	3	Yes
I_4	P	4	Yes
23	P	4	Yes
24	P	4	Yes
54	P	4	Yes
55	P	4	Yes
56	P	2	Yes
64	P	4	Yes
65	P	4	Yes
66	P	4	Yes
67	P	4	Yes
117	P	3	Yes
122	P	3	Yes
I_5	P	3	Yes
128	I_5	4	Yes
I_1	2	3	Yes
2	7	2	Yes
2	12	3	Yes
2	20	2	Yes
2	22	3	Yes
2	I_4	3	Yes
2	I_3	3	Yes
2	23	3	Yes
2	24	3	Yes
128	122	4	Yes
3	24	3	Yes
3	27	3	Yes
121	122	2	Yes
I_1	I_2	3	Yes
I_1	20	3	Yes

I_1	I_3	4	Yes
I_1	27	4	Yes
I_1	40	3	Yes
5	I_4	3	Yes
5	23	3	Yes
109	122	3	Yes
7	14	3	Yes
7	I_4	3	Yes
7	23	3	Yes
7	24	3	Yes
7	49	2	Yes
99	117	3	Yes
99	I_5	3	Yes
I_2	I_4	3	Yes
I_2	I_3	1	Yes
I_2	27	3	Yes
I_2	55	3	Yes
I_2	57	4	Yes
8	57	4	Yes
8	60	2	Yes
8	64	3	Yes
8	65	3	Yes
8	66	3	Yes
8	67	3	Yes
12	14	2	Yes
12	128	3	Yes
14	55	4	Yes
14	121	3	Yes
71	117	4	Yes
20	I_4	3	Yes
20	109	3	Yes
20	122	4	Yes
20	I_5	4	Yes
20	128	4	Yes
22	I_4	2	Yes
22	117	3	Yes
I_3	I_4	2	Yes
49	I_4	3	Yes
60	I_4	3	Yes
I_4	121	3	Yes
I_4	122	3	Yes
I_4	I_5	3	Yes
I_3	128	3	Yes
23	34	3	Yes
23	71	3	Yes
23	109	2	Yes

23	117	3	Yes
23	121	3	Yes
23	122	3	Yes
23	128	3	Yes
60	65	3	Yes
60	67	3	Yes
24	71	3	Yes
24	99	2	Yes
24	117	3	Yes
24	I_5	3	Yes
24	128	3	Yes
57	66	3	Yes
27	I_5	3	Yes
128	55	3	Yes
54	56	2	Yes
65	54	4	Yes
49	117	4	Yes
34	64	3	Yes
34	122	4	Yes
40	55	3	Yes
65	36	2	Yes
36	122	3	Yes
P	12	4	No
P	14	4	No
P	I_4	4	No
P	23	4	No
P	27	4	No
P	55	4	No
P	66	4	No
P	67	4	No
P	73	2	No
P	76	3	No
P	77	3	No
P	117	3	No
P	121	3	No
P	122	3	No
P	I_5	3	No
P	128	3	No
3	1	4	No
I_2	1	4	No
1	12	1	No
1	14	3	No
14	1	3	No
1	27	3	No
27	1	3	No
54	1	3	No

56	1	3	No
1	57	4	No
57	1	4	No
I_5	128	4	No
2	3	4	No
3	2	4	No
2	I_1	3	No
5	2	2	No
7	2	2	No
2	I_2	4	No
8	2	4	No
10	2	4	No
19	2	2	No
20	2	2	No
22	2	3	No
23	2	3	No
24	2	3	No
122	I_5	2	No
I_5	122	2	No
122	128	4	No
10	3	4	No
24	3	3	No
26	3	2	No
122	121	2	No
121	I_5	4	No
I_5	121	4	No
121	128	4	No
128	121	4	No
I_2	I_1	3	No
8	I_1	3	No
20	I_1	3	No
38	I_1	2	No
54	I_1	4	No
56	I_1	4	No
117	121	4	No
117	122	4	No
122	117	4	No
117	I_5	4	No
I_5	117	4	No
117	128	2	No
5	7	2	No
7	5	2	No
5	I_2	4	No
5	22	3	No
5	I_3	3	No
109	121	3	No

121	109	3	No
122	109	3	No
109	128	3	No
128	109	3	No
7	I_2	4	No
10	7	4	No
7	22	3	No
22	7	3	No
7	I_3	3	No
24	7	3	No
38	7	3	No
49	7	2	No
99	109	4	No
117	99	3	No
99	136	4	No
136	99	4	No
8	I_2	4	No
22	I_2	3	No
I_4	I_2	3	No
38	I_2	3	No
54	I_2	3	No
56	I_2	3	No
57	I_2	4	No
60	I_2	4	No
77	128	4	No
128	77	4	No
8	20	2	No
60	8	2	No
65	8	3	No
76	77	2	No
77	76	2	No
10	23	3	No
10	24	3	No
10	40	2	No
10	55	3	No
10	56	3	No
56	10	3	No
10	71	2	No
75	76	4	No
75	77	4	No
75	117	4	No
14	12	2	No
15	12	4	No
22	12	4	No
I_4	12	4	No
I_3	12	4	No

23	12	4	No
24	12	4	No
27	12	4	No
54	12	4	No
56	12	4	No
117	12	3	No
122	12	3	No
I_5	12	3	No
128		3	No
74	76	3	No
74	77	3	No
22	14	4	No
I_4	14	4	No
I_3	14	4	No
23	14	4	No
24	14	4	No
55	14	4	No
56	14	4	No
117	14	3	No
121	14	3	No
14	128	3	No
128	14	3	No
73	76	3	No
76	73	3	No
99	73	4	No
73	109	4	No
109	73	4	No
117	73	3	No
73	121	3	No
121	73	3	No
73	122	3	No
122	73	3	No
I_5	73	3	No
73	128	3	No
128	73	3	No
136	73	4	No
65	15	4	No
15	66	4	No
66	15	4	No
15	67	4	No
67	15	4	No
71	73	3	No
117	71	4	No
71	128	4	No
19	20	2	No
19	22	3	No

19	I_4	3	No
19	28	3	No
28	19	3	No
19	49	2	No
19	70	4	No
19	99	3	No
19	117	4	No
19	122	4	No
19	I_5	4	No
70	71	4	No
70	99	3	No
99	70	3	No
70	117	4	No
20	I_3	3	No
28	20	3	No
20	49	4	No
49	20	4	No
74	67	4	No
67	76	3	No
67	77	3	No
67	I_5	3	No
I_5	67	3	No
22	I_3	2	No
22	49	3	No
49	22	3	No
66	67	4	No
67	66	4	No
74	66	4	No
75	66	3	No
66	76	3	No
66	77	3	No
66	128	3	No
I_4	I_3	2	No
I_5	I_4	3	No
65	66	4	No
65	67	2	No
65	74	4	No
74	65	4	No
65	76	3	No
65	77	3	No
65	122	3	No
I_3	27	4	No
27	I_3	4	No
38	I_3	4	No
54	I_3	4	No
I_3	55	4	No

55	I_3	4	No
56	I_3	4	No
128	I_3	3	No
65	64	2	No
64	66	2	No
66	64	2	No
64	67	4	No
67	64	4	No
74	64	4	No
64	76	3	No
64	77	3	No
64	109	2	No
23	24	2	No
24	23	2	No
34	23	3	No
54	23	4	No
56	23	4	No
23	64	4	No
64	23	4	No
117	23	3	No
65	60	3	No
24	27	4	No
56	24	4	No
99	24	2	No
66	57	3	No
26	34	4	No
26	I_5	4	No
26	136	3	No
56	57	3	No
56	64	4	No
56	66	2	No
56	74	4	No
74	56	4	No
56	77	3	No
56	117	3	No
56	128	1	No
54	27	2	No
27	55	4	No
55	27	4	No
56	27	2	No
27	57	3	No
57	27	3	No
66	27	4	No
67	27	4	No
I_5	27	3	No
27	128	3	No

128	27	3	No
56	55	2	No
66	55	4	No
55	128	3	No
28	30	3	No
28	73	4	No
28	136	4	No
54	55	4	No
56	54	2	No
54	57	3	No
54	64	4	No
54	65	4	No
54	66	4	No
54	122	3	No
54	128	3	No
30	34	4	No
34	30	4	No
30	49	4	No
49	30	4	No
30	136	3	No
136	30	3	No
49	70	4	No
70	49	4	No
49	99	3	No
99	49	3	No
49	121	4	No
64	34	3	No
34	109	3	No
34	121	4	No
34	136	3	No
55	40	3	No
56	40	3	No
70	40	4	No
40	71	4	No
71	40	4	No
40	77	4	No
40	128	4	No
54	36	4	No
36	64	4	No
64	36	4	No
36	66	4	No
36	67	4	No
38	40	3	No
38	55	4	No
38	56	4	No

Table S6. The 91 predicted reaction paths of the cobalt-catalyzed hydroformylation.

**Paths**	**Sum of chemical distances**
R→2→24→P	8
R→2→23→P	8
R→2→I_4→P	8
R→3→24→P	10
R→5→23→P	10
R→5→I_4→P	10
R→7→24→P	10
R→7→23→P	10
R→7→I_4→P	10
R→2→7→24→P	10
R→2→7→23→P	10
R→2→7→I_4→P	10
R→I_2→55→P	10
R→I_2→I_4→P	10
R→I_2→I_3→I_4→P	10
R→8→67→P	10
R→8→66→P	10
R→8→65→P	10
R→8→64→P	10
R→2→20→I_4→P	10
R→2→20→I_5→P	10
R→2→20→122→P	10
R→2→22→I_4→P	10
R→2→22→117→P	10
R→2→I_3→I_4→P	10
R→2→24→117→P	10
R→2→23→117→P	10
R→2→23→122→P	10
R→2→I_4→122→P	10
R→2→24→I_5→P	10
R→2→I_4→I_5→P	10
R→I_1→I_2→I_3→I_4→P (Heck-Breslow mechanism)	12
R→I_1→40→55→P	12
R→I_1→I_2→55→P	12
R→I_1→2→24→P	12
R→I_1→I_2→I_4→P	12
R→I_1→20→I_4→P	12
R→I_1→2→23→P	12
R→I_1→I_3→I_4→P	12
R→I_1→20→I_5→P	12

R→I_1→2→I_4→P	12
R→I_1→27→I_5→P	12
R→I_1→20→122→P	12
R→I_2→27→I_5→P	12
R→3→27→I_5→P	12
R→7→49→I_4→P	12
R→7→49→117→P	12
R→2→7→49→I_4→P	12
R→2→7→49→117→P	12
R→8→60→67→P	12
R→8→60→65→P	12
R→8→60→I_4→P	12
R→2→24→99→I_5→P	12
R→2→24→99→117→P	12
R→2→20→109→122→P	12
R→2→23→109→122→P	12
R→2→23→121→122→P	12
R→2→I_4→121→122→P	12
R→2→12→14→55→P	14
R→2→12→128→55→P	14
R→2→12→14→121→122→P	14
R→2→12→128→I_5→P	14
R→2→12→128→122→P	14
R→7→14→55→P	14
R→7→14→121→122→P	14
R→2→7→14→55→P	14
R→2→7→14→121→122→P	14
R→2→23→34→64→P	14
R→2→23→34→122→P	14
R→8→65→36→122→P	14
R→8→65→54→P	14
R→8→65→54→56→P	14
R→8→57→66→P	14
R→I_2→57→66→P	14
R→2→24→71→117→P	14
R→2→23→71→117→P	14
R→2→20→128→55→P	14
R→2→20→128→I_5→P	14
R→2→20→128→122→P	14
R→I_2→I_3→128→55→P	14
R→I_2→I_3→128→I_5→P	14
R→I_2→I_3→128→122→P	14
R→2→24→128→55→P	14

R→2→24→128→I_5→P	14
R→2→24→128→122→P	14
R→2→23→128→55→P	14
R→2→23→128→I_5→P	14
R→2→23→128→122→P	14
R→2→I_3→128→55→P	14
R→2→I_3→128→I_5→P	14
R→2→I_3→128→122→P	14

2. DFT-optimized Cartesian coordinates of intermediates and transition states

The following is the Cartesian coordinates of intermediates and transition states in the paths shown in Fig. 9:

Structure $Co(CO)_3H$
Co 0.000001 -0.245699 -0.000374
C -1.746218 -0.618395 0.000124
O -2.835524 -0.941438 0.000447
C -0.000003 1.555500 -0.000388
O -0.000011 2.692672 0.000434
C 1.746222 -0.618389 0.000124
O 2.835530 -0.941426 0.000447
H 0.000002 -1.756881 0.000321

Structure C_2H_4
C 0.000000 0.000000 0.661568
C 0.000000 0.000000 -0.661568
H 0.000000 0.923204 1.233890
H 0.000000 -0.923204 1.233890
H 0.000000 0.923204 -1.233890
H 0.000000 -0.923204 -1.233890

Structure CO
C 0.000000 0.000000 -0.643619
O 0.000000 0.000000 0.482714

Structure H_2
H 0.000000 0.000000 0.373292
H 0.000000 0.000000 -0.373292

Structure I_1
Co -0.000147 -0.129207 -0.143279
C 0.000672 -1.907365 1.176382
C 0.002868 -0.807957 1.978537
H -0.920305 -2.435300 0.952680

H 0.920106 -2.436666 0.949599
H -0.918117 -0.425544 2.406987
H 0.925966 -0.426872 2.403614
H -0.000257 -1.315619 -1.054615
C -1.648101 -0.106041 -0.826883
O -2.670855 -0.126293 -1.332458
C -0.000556 1.539409 0.526725
O -0.000883 2.607107 0.923544
C 1.647286 -0.104977 -0.828324
O 2.669684 -0.124541 -1.334630

Structure T_1
C 0.000013 -1.876443 1.211191
C -0.000003 -0.720890 1.977732
H -0.915265 -2.452346 1.106800
H 0.915312 -2.452312 1.106794
H -0.920745 -0.369246 2.432120
H 0.920731 -0.369222 2.432120
H -0.000010 -1.577124 -0.508481
Co -0.000003 -0.146407 -0.058172
C -1.589703 -0.120051 -0.908724
O -2.571385 -0.118417 -1.490495
C 0.000007 1.536939 0.537615
O 0.000018 2.608879 0.924360
C 1.589692 -0.120059 -0.908736
O 2.571370 -0.118428 -1.490513

Structure I_2
Co -0.004022 0.000274 0.098890
C 1.646326 0.030090 1.625559
C 1.967594 0.038185 0.166687
H 1.965872 0.930858 2.153395
H 1.999183 -0.860000 2.150295
H 2.448973 0.942319 -0.201386
H 2.483208 -0.846074 -0.203466
H 0.510994 0.008745 1.831278
C -1.749502 -0.034612 0.527687
O -2.854550 -0.056315 0.801411
C 0.019352 1.610237 -0.693849
O 0.057655 2.591973 -1.273707
C 0.082273 -1.607017 -0.695465
O 0.159906 -2.586225 -1.275688

Structure T_2
Co -0.540744 0.080158 0.156224
C -0.838445 -1.878100 -0.111356
C 0.013226 1.794578 0.165454

C 0.063130 -0.370588 1.777096
C -2.006244 0.301812 -0.844349
C 0.400840 -2.050463 -0.974537
H -1.752386 -2.135272 -0.652257
H -0.806376 -2.501947 0.784685
O 0.382519 2.870708 0.159329
O 0.344304 -0.679383 2.837129
O -2.999452 0.415039 -1.390521
H 0.527924 -1.228537 -1.699686
H 0.351414 -2.966222 -1.578879
H 1.316026 -2.105373 -0.376148
C 2.858811 0.412330 -0.797413
O 3.774575 -0.166906 -1.104078

Structure I_3
Co 0.234723 -0.000002 0.006986
C -0.291047 -1.588121 -0.696349
O -0.668611 -2.586782 -1.091627
C -2.833578 0.001011 -0.109026
C -1.668357 -0.002394 0.856432
H -2.843642 -0.878234 -0.760656
H -3.781533 -0.000774 0.445137
H -1.704256 -0.885996 1.501510
H -1.703751 0.877085 1.507121
H -2.843417 0.884649 -0.754700
C 1.886143 0.001770 -0.743670
O 2.917648 0.002620 -1.218784
C -0.290396 1.591828 -0.688308
O -0.667046 2.592903 -1.078330
C 0.785709 -0.004665 1.743296
O 1.044037 -0.007895 2.851079

Structure T_3
C 1.121231 -1.106234 0.072572
O 1.927659 -1.907285 0.278304
C 2.620293 1.196198 -0.269929
C 1.610091 0.401629 -1.064528
H 3.177709 0.563926 0.428573
H 3.352106 1.666633 -0.937642
H 2.079468 -0.343710 -1.711480
H 1.021630 1.030549 -1.735018
H 2.140844 1.989581 0.310577
Co -0.265284 -0.047795 -0.006972
C -0.621213 -0.125186 1.754919
O -0.853398 -0.210006 2.866597
C -0.949691 1.625023 -0.264359
O -1.332633 2.683227 -0.427017

C -1.392169 -1.060762 -1.039096
O -2.109173 -1.716001 -1.630912

Structure I_4
Co -0.389332 -0.011985 -0.028710
C 1.325988 0.661022 0.119316
O 2.091422 1.258350 0.794960
C 2.760139 -0.966158 -1.133524
C 1.608492 0.017458 -1.253626
H 3.648009 -0.457311 -0.748358
H 3.003754 -1.408716 -2.102623
H 1.826265 0.845349 -1.935926
H 0.713566 -0.490113 -1.681435
H 2.510181 -1.777339 -0.442773
C -0.121353 -1.040306 1.416836
O -0.003540 -1.629323 2.385079
C -1.827779 -0.890928 -0.759316
O -2.723977 -1.430858 -1.203265
C -1.066865 1.646220 0.035285
O -1.521598 2.682815 0.165284

Structure T_4
Co -0.591957 0.023392 -0.002768
C -0.618854 -1.685559 -0.546455
C -0.367605 1.734302 -0.492365
C -2.321224 0.142484 0.550648
C 1.343026 -0.105690 -0.335050
O -0.660181 -2.733927 -0.987738
O -0.258944 2.789296 -0.904681
O -3.400458 0.216896 0.899053
O 1.948137 -0.096624 -1.363914
C 2.014668 -0.224643 1.030968
C 3.501956 0.074284 0.985481
H 1.825014 -1.251161 1.376252
H 1.491334 0.425663 1.745063
H 3.951620 -0.049291 1.974153
H 3.686200 1.098845 0.650607
H 4.009134 -0.593102 0.284935
H -0.656670 -0.268009 2.922241
H -0.664015 -1.010720 2.820378

Structure I_5'
Co -0.608855 0.000206 -0.283547
H -0.252191 -0.418118 -1.854632
H -0.251666 0.417366 -1.855059
C 1.360552 -0.000018 0.167837
O 1.757988 0.000628 1.294061

C 3.772080 -0.001124 -0.627521
C 2.309774 -0.001058 -1.022585
H 4.409441 -0.001043 -1.516080
H 4.018267 0.878702 -0.027611
H 2.055067 -0.874090 -1.638012
H 2.055550 0.871216 -1.639270
H 4.018314 -0.881047 -0.027778
C -0.569384 -1.599040 0.552343
O -0.541568 -2.587863 1.114663
C -0.568668 1.600067 0.551300
O -0.539980 2.589133 1.113120
C -2.418323 -0.000092 -0.602778
O -3.542676 -0.000770 -0.759015

Structure T_5
Co 0.601617 -0.083405 -0.240254
C 0.938623 1.640048 0.323523
O 1.125601 2.717616 0.613917
C 0.346811 -1.156364 1.208425
O 0.140206 -1.863474 2.073277
C 2.329609 -0.540454 -0.692079
O 3.359527 -0.852736 -1.047831
H 0.328839 0.273937 -1.659193
H 0.200157 -1.060420 -1.279000
C -1.368133 0.307546 -0.089960
O -1.762780 1.207140 0.591535
C -3.779504 -0.289566 -0.615071
C -2.315952 -0.602420 -0.848337
H -4.413102 -0.967564 -1.193462
H -4.043882 -0.391651 0.440811
H -2.044080 -0.524914 -1.909048
H -2.066069 -1.634850 -0.569343
H -4.014687 0.736288 -0.910099

Structure I_5
Co 0.594656 -0.106358 -0.193574
C -1.370973 0.295220 -0.145836
O -1.765305 1.308626 0.352530
C -3.776023 -0.357743 -0.637485
C -2.312149 -0.733642 -0.737093
H -4.402111 -1.137681 -1.079579
H -4.081530 -0.223183 0.403469
H -1.992927 -0.888847 -1.775519
H -2.097947 -1.686847 -0.234213
H -3.979896 0.581036 -1.159284
H 0.294289 0.355868 -1.575309
H 0.188436 -1.352813 -0.874076

C 0.964724 1.642682 0.263480
O 1.175486 2.731990 0.478563
C 0.411896 -0.976720 1.394082
O 0.239235 -1.572168 2.344825
C 2.259890 -0.624398 -0.780837
O 3.219556 -0.999480 -1.253026

Structure T_6
Co 0.592889 -0.144854 -0.134379
C 1.059973 1.576101 0.334159
O 1.345005 2.636304 0.604629
C 0.456583 -0.929152 1.488012
O 0.323842 -1.484359 2.471441
C 2.140869 -0.645095 -0.964763
O 3.044754 -1.023788 -1.537874
H -0.180382 0.309044 -1.354991
H 0.189597 -1.485026 -0.616157
C -1.373915 0.369674 -0.316881
O -1.729397 1.480980 -0.048776
C -3.798384 -0.336203 -0.525571
C -2.345604 -0.761920 -0.578327
H -4.453371 -1.188817 -0.723988
H -4.056301 0.072886 0.454563
H -2.077676 -1.195437 -1.550567
H -2.129877 -1.553774 0.151206
H -4.010769 0.438647 -1.266955

Structure P'
Co -0.928627 0.020030 -0.294051
H 0.650258 0.835198 0.566392
H -0.576010 0.677108 -1.603087
C 1.716986 0.450069 0.709600
O 1.991814 -0.221407 1.659639
C 4.084317 0.441837 -0.160920
C 2.655101 0.891143 -0.367387
H 4.723987 0.788540 -0.975924
H 4.149386 -0.648280 -0.113799
H 2.565245 1.984976 -0.440038
H 2.230962 0.523045 -1.314034
H 4.485949 0.829907 0.778954
C -1.475001 -0.816318 1.220231
O -1.838037 -1.345800 2.156146
C -0.361285 -1.376008 -1.246441
O -0.052285 -2.223803 -1.940654
C -2.003343 1.439346 -0.291287
O -2.708680 2.327044 -0.392866

Structure CH_3CH_2CHO
H 1.584155 1.318788 0.000052
C 0.922950 0.418772 0.000023
O 1.393887 -0.684752 -0.000016
C -1.430461 -0.499354 0.000010
C -0.543703 0.726143 -0.000023
H -2.487233 -0.221419 -0.000297
H -1.237859 -1.120224 0.878918
H -0.732711 1.374035 -0.868946
H -0.732705 1.374054 0.868890
H -1.237455 -1.120588 -0.878553

Structure T_7
C 2.476294 0.395055 0.983116
C 1.798204 0.419401 -0.378105
H 2.054166 1.138130 1.669001
H 3.547820 0.618589 0.891687
H 1.933397 1.395749 -0.853810
H 2.265100 -0.302946 -1.055424
H 2.394742 -0.581906 1.473853
Co -0.164195 -0.020097 -0.037968
C -1.889331 -0.408992 0.283391
O -2.986964 -0.654575 0.460345
C -0.393736 1.724121 -0.365041
O -0.500140 2.821215 -0.647319
C 0.382366 -1.686665 -0.403119
O 0.770612 -2.712515 -0.705205
H -0.084792 0.359800 2.715239
H -0.188026 -0.375306 2.600565

Structure 27'
Co -0.185263 0.013754 -0.267161
C 1.690176 0.843702 -0.086030
C -1.844908 -0.715707 -0.416975
C -0.716499 1.584566 0.429080
C 0.536141 -1.316928 0.700248
C 2.783149 0.060939 -0.784170
H 1.919493 0.973431 0.976762
H 1.613277 1.845870 -0.521539
O -2.881959 -1.174065 -0.488888
O -0.977749 2.581394 0.914424
O 1.027811 -2.098924 1.367848
H 2.920726 -0.941888 -0.366500
H 3.746590 0.580369 -0.691942
H 2.586038 -0.057679 -1.855172
H -0.065393 0.394883 -1.891835
H 0.248178 -0.373033 -1.836411

Structure T_8
Co -0.188787 0.032631 -0.218470
C 1.794896 0.935898 -0.372039
C -1.664865 -0.873658 -0.608713
C -0.929340 1.572681 0.486021
C 0.477871 -1.179140 0.952456
C 2.947340 0.045212 -0.789067
H 1.820646 1.157961 0.693826
H 1.820298 1.902602 -0.887286
O -2.606331 -1.448721 -0.892103
O -1.406729 2.502903 0.936318
O 0.915909 -1.931950 1.689372
H 2.965350 -0.888280 -0.219483
H 3.905706 0.551229 -0.624343
H 2.895236 -0.224800 -1.849089
H -0.083549 0.134890 -1.775174
H 0.795371 0.501547 -1.320402

Structure P_{side}
Co -0.488732 -0.301876 -0.152418
C 1.636449 1.751703 -0.907109
C 0.552597 -1.747149 -0.230766
C -2.047403 0.474728 -0.530074
C -0.251562 0.206753 1.563050
C 2.885322 1.122933 -0.322223
H 1.209764 2.510109 -0.243603
H 1.832371 2.228274 -1.872755
O 1.148909 -2.710214 -0.338210
O -3.069631 0.883357 -0.817531
O -0.108238 0.515117 2.648366
H 2.693244 0.679696 0.660667
H 3.680722 1.863368 -0.194476
H 3.276126 0.330493 -0.968488
H -0.767569 -0.815227 -1.542903
H 0.850361 0.994042 -1.101442

Structure I_{4-2}
Co 0.601797 -0.000102 -0.101016
C 0.515248 1.599378 0.694267
O 0.463387 2.562020 1.306479
C 2.355305 -0.000229 -0.612825
O 3.434153 -0.000151 -0.968175
C 0.514866 -1.599486 0.694137
O 0.462667 -2.562213 1.306212
C -1.245284 0.000217 -0.408153
O -1.155709 0.000619 -1.609037

C -2.493912 0.000280 0.405940
H -2.422982 -0.867964 1.075609
H -2.423095 0.869313 1.074663
C -3.770496 -0.000156 -0.412175
H -3.822481 -0.881249 -1.056910
H -4.647192 -0.000566 0.239852
H -3.823141 0.880989 -1.056775

Structure T_{4-2}
Co 0.685992 -0.000434 -0.217203
C 2.065116 0.003775 1.097155
O 2.796974 0.006360 1.963138
C 0.347003 1.602358 -0.938893
O 0.126745 2.588444 -1.474000
C 0.348667 -1.607504 -0.929812
O 0.129781 -2.596904 -1.459355
H 2.356918 -0.003797 -2.082080
H 3.060041 -0.006080 -1.813624
C -1.028633 0.001206 0.572844
O -0.896523 0.006329 1.757734
C -2.339208 -0.003350 -0.170320
H -2.323171 0.861599 -0.845302
H -2.322998 -0.876919 -0.834154
C -3.552213 0.002205 0.739267
H -3.561041 0.887851 1.379628
H -4.471076 -0.001736 0.147519
H -3.560688 -0.875171 1.390907

Structure I_{5-2}'
Co -0.933786 0.149731 0.019853
C -1.002428 -0.794374 1.581723
O -1.016633 -1.377193 2.558681
C -1.042827 -0.599511 -1.641503
O -1.078946 -1.062883 -2.679791
C -0.460712 1.908419 0.101772
O -0.195936 3.016130 0.151726
H -2.453631 0.880368 0.130399
H -2.603245 0.079379 0.006916
C 0.958277 -0.513306 -0.038640
O 1.166371 -1.684585 -0.124523
C 2.079880 0.506211 0.034242
H 1.918816 1.225381 -0.779341
H 1.935060 1.078118 0.960257
C 3.461905 -0.111507 -0.030859
H 3.598979 -0.673679 -0.958073
H 4.229006 0.666037 0.017052
H 3.623818 -0.805680 0.797593

Structure T_{5-2}
Co -0.884933 -0.000169 -0.331665
C -0.660089 -1.749969 -0.629789
O -0.554020 -2.858576 -0.857023
C -0.661810 1.750102 -0.628373
O -0.556939 2.859006 -0.854768
C -1.395022 -0.001156 1.424924
O -1.774924 -0.001831 2.495425
H -2.326094 -0.000760 -0.788870
H -1.336354 0.000143 -1.745984
C 1.185767 0.000737 -0.359032
O 1.785156 0.001835 -1.387697
C 1.854896 -0.000092 0.999655
H 1.463982 0.870326 1.545349
H 1.464637 -0.871692 1.543929
C 3.368927 0.000537 0.938361
H 3.739883 0.881333 0.407865
H 3.796376 -0.000295 1.944544
H 3.740558 -0.878927 0.406137

Structure I_{5-2}
Co -0.871807 -0.000226 -0.234718
C -0.856679 -1.745068 -0.594230
O -0.894303 -2.841432 -0.886332
C -0.857999 1.744723 -0.593800
O -0.896600 2.841187 -0.885388
C -1.082615 -0.000484 1.577222
O -1.286284 -0.000788 2.693163
H -2.388736 -0.000793 -0.279140
H -1.004404 -0.000092 -1.707643
C 1.175654 0.000528 -0.435514
O 1.680044 0.001127 -1.517032
C 1.990924 0.000311 0.844390
H 1.663719 0.872262 1.428723
H 1.664016 -0.872046 1.428278
C 3.489705 0.000633 0.624480
H 3.803927 0.880996 0.057975
H 4.019231 0.000476 1.581083
H 3.804234 -0.879328 0.057521

Structure T_{6-2}
Co 0.759406 -0.224730 -0.179857
C 1.769512 1.200095 -0.582835
O 2.441376 2.067439 -0.870276
C 0.417236 -1.919971 -0.583604
O 0.247549 -3.007641 -0.874406

C 0.734063 -0.197916 1.658818
O 0.727486 -0.191957 2.794315
H 2.112028 -0.877441 -0.387715
H -0.069965 0.157219 -1.380368
C -1.088613 0.698680 -0.261246
O -1.166989 1.889572 -0.226289
C -2.284480 -0.222634 -0.154070
H -2.265815 -0.873556 -1.039327
H -2.104884 -0.894911 0.695996
C -3.604320 0.508614 -0.020539
H -3.781415 1.161483 -0.879138
H -4.431603 -0.202666 0.047238
H -3.618068 1.137064 0.873543

Structure P”
Co -0.892032 0.120207 0.109418
C -1.960690 -1.019337 -0.816883
O -2.700293 -1.706537 -1.342448
C -0.944553 1.895951 -0.207890
O -1.051854 3.021586 -0.359767
C -0.333893 -0.468669 1.733302
O -0.087068 -0.811021 2.791793
H -2.119481 0.416183 0.830756
H 0.374516 -0.123463 -1.042119
C 1.426343 -0.514394 -0.697697
O 1.654666 -1.678728 -0.769294
C 2.395177 0.584655 -0.407068
H 2.281986 1.330829 -1.207398
H 2.028158 1.094514 0.495454
C 3.822197 0.104811 -0.258936
H 4.165382 -0.396630 -1.167645
H 4.491380 0.943855 -0.056308
H 3.911821 -0.611374 0.561741

Structure 109
Co -0.327206 -0.026165 -0.218529
C -1.990999 -0.771476 0.055535
O -2.999603 -1.221965 0.314190
C -0.868709 1.648619 -0.450607
O -1.184417 2.724292 -0.645829
C 0.549496 -1.572579 -0.493206
O 1.090153 -2.540764 -0.732448
C 0.119063 0.083503 1.775089
H -0.370312 0.947042 2.230738
H -0.139476 -0.818423 2.331618
C 1.601615 0.289705 1.514184
H 2.128809 -0.672441 1.493467

H 2.137357 0.941578 2.219947
C 1.537836 0.816883 0.106483
O 2.569133 0.373176 -0.706142
H 2.462239 0.742538 -1.587269
H 1.445561 1.908902 0.072499
H -0.321554 0.011408 -1.743739

Structure 121
Co 0.378679 -0.020164 -0.027374
C 0.767872 1.738291 0.017374
O 1.025520 2.841336 0.149595
C 1.918421 -0.605354 -0.777809
O 2.886971 -0.975070 -1.248760
C -1.542672 0.338543 0.294300
H -1.995955 -0.251249 1.098576
O -2.035569 1.639907 0.269739
H -1.964683 2.022997 1.147722
C -1.610620 -0.306571 -1.046351
H -2.030123 0.411749 -1.758769
H -0.552351 -0.462945 -1.485772
C -2.310546 -1.653149 -1.085644
H -1.897641 -2.329096 -0.329852
H -3.378892 -1.532034 -0.882382
H -2.203263 -2.137563 -2.060129
C 0.383588 -1.095534 1.396482
O 0.393369 -1.715522 2.356875

Structure 122
Co 0.636389 0.000080 0.154874
H 0.868413 0.001458 1.680035
C 0.567169 1.522783 -0.817148
O 0.498406 2.483011 -1.431380
C 0.566779 -1.523269 -0.816000
O 0.497867 -2.483969 -1.429486
C -1.206353 0.000365 0.542674
O -1.678548 0.001726 1.761560
H -0.916119 0.002363 2.362537
C -2.313026 -0.000672 -0.461770
H -2.128551 0.861384 -1.118558
H -2.128425 -0.863893 -1.116978
C -3.737594 -0.000298 0.059800
H -3.941129 -0.881407 0.673630
H -3.941183 0.881774 0.672226
H -4.441982 -0.000990 -0.775643
C 2.354566 -0.000128 0.594768
O 3.439431 -0.000210 0.952707

Structure 14

Co 0.528967 0.015778 0.100440
C 1.663123 -1.182346 -0.599135
O 2.467017 -1.905971 -0.957503
C -0.278312 -1.087144 1.318560
O -0.802710 -1.763704 2.063812
C 0.856701 1.135833 -1.410358
H 1.774350 1.673328 -1.736826
O -0.168711 1.193788 -2.023373
H -3.202480 -0.985782 -1.880167
C -2.716276 -0.854554 -0.907037
H -3.020606 -1.689627 -0.266912
H -1.634443 -0.951152 -1.081852
C -3.070501 0.484158 -0.287087
H -2.728010 1.315069 -0.912338
H -4.151497 0.591001 -0.153225
H -2.611697 0.607261 0.702397
C 0.649631 1.479260 1.128302
O 0.837665 2.371218 1.811760

Structure I_{4-3}

Co 0.403482 0.013248 -0.046416
C -1.549578 0.074390 -0.359414
O -2.154928 0.046261 -1.387214
H -0.401732 -0.022268 2.181808
C 0.330482 1.752834 -0.467362
O 0.316849 2.829717 -0.836790
C 0.276345 -1.665582 -0.670019
O 0.220833 -2.688367 -1.164671
C 2.148683 -0.045487 0.463960
O 3.237804 -0.081837 0.786797
C -2.217869 0.207623 1.002132
H -3.233840 -0.197420 0.922305
H -2.321317 1.291059 1.159782
C -1.429461 -0.418145 2.140472
H -1.365238 -1.505172 2.036210
H -1.887959 -0.203875 3.109538

Structure 20

C -2.060770 -0.265077 -0.409711
O -3.105827 -0.579723 -0.717468
H -0.088225 0.314508 -1.576710
Co -0.266351 0.069098 -0.110639
C 1.671869 -0.042214 -0.503373
O 2.516312 0.791143 -0.542916
C -0.010257 -0.443269 1.625745
O 0.165178 -0.807783 2.686829

C -0.240135 1.888729 0.034296
O -0.140369 3.016158 0.044979
C 1.704413 -1.517952 -0.716428
H 2.251793 -1.799803 -1.626800
H 2.234318 -1.966414 0.135943
C 0.205520 -1.812021 -0.701687
H -0.208600 -2.028998 -1.685952
H -0.103988 -2.592474 -0.003675

Structure 23
Co -0.023651 0.000042 -0.144118
C -1.664310 0.002547 -0.837449
O -2.678805 0.004676 -1.359299
C -0.106322 1.493118 0.881818
O -0.160613 2.432697 1.524380
C 1.811300 -0.004028 -0.254321
H 2.477655 -0.009032 0.624435
O 2.577067 -0.000927 -1.307869
C -0.110980 -1.494096 0.879563
O -0.168734 -2.434858 1.520113
H 0.023134 0.006223 -1.674778
H 2.008353 0.003713 -2.094728

Structure 24
Co 0.000017 -0.366686 -0.268491
C -0.000026 0.027628 1.711934
O -0.000110 1.122144 2.184935
H 0.000030 -0.873089 2.378664
C 1.751143 -0.695781 -0.257837
O 2.849347 -0.979303 -0.284751
C -0.000005 1.400621 -0.774703
O -0.000023 2.480531 -1.105368
H 0.000045 -0.775746 -1.736611
C -1.751099 -0.695830 -0.257890
O -2.849295 -0.979384 -0.284827
H 0.000029 -1.822370 -0.001729

Structure 24'
Co 0.065262 0.002264 0.254951
C 1.902712 -0.036146 0.432580
O 3.033085 -0.061048 0.518999
C -0.000134 1.610982 -0.555282
O -0.055958 2.604894 -1.107790
C -1.911582 0.035615 0.059585
H -2.304595 0.080960 -0.986738
O -2.653858 0.007789 0.989027
C -0.063954 -1.595179 -0.568603

O -0.154709 -2.581700 -1.129761
H -0.169971 0.416696 1.866606
H -0.198238 -0.409914 1.862979

Structure 3
Co -0.000681 -0.000591 0.510274
H 0.999133 0.257936 1.561241
H -0.727265 0.735896 1.559101
H -0.278282 -0.997776 1.558944
C -1.609180 -0.417850 -0.228807
O -2.646547 -0.686903 -0.598720
C 1.167457 -1.182950 -0.227980
O 1.920690 -1.945261 -0.597955
C 0.443005 1.601299 -0.227099
O 0.727998 2.634277 -0.597497

Structure 3'
Co 0.000332 -0.165296 0.443081
H 0.418631 0.093690 2.028830
H -0.417254 0.091886 2.029405
H 0.003012 -1.619175 0.767009
C -1.586589 -0.667300 -0.215819
O -2.567059 -1.054427 -0.645956
C 1.587625 -0.664719 -0.216293
O 2.568564 -1.050966 -0.646209
C -0.001639 1.577905 -0.075603
O -0.002721 2.658052 -0.425603

Structure 5
Co 0.729596 -0.000072 0.135431
C 0.563765 1.463602 -0.913285
O 0.376859 2.385799 -1.559265
C 0.564736 -1.464271 -0.912785
O 0.378370 -2.386770 -1.558474
C -0.479021 -0.000122 1.270842
O -1.532608 -0.000039 1.966102
H -2.349829 -0.000149 1.400896
C 2.495199 0.000600 0.594759
O 3.576162 0.001155 0.951626
C -3.170114 0.000903 -0.675890
H -2.747761 0.924681 -1.063224
H -2.746845 -0.921469 -1.065554
C -4.152679 -0.000661 0.218725
H -4.588488 0.923366 0.589715
H -4.587743 -0.925945 0.587426

Structure 7

Co 0.007358 0.028822 0.096290
C -0.424635 -1.696921 0.521822
O -0.690038 -2.755373 0.833448
C 1.594272 -0.260958 -0.685424
O 2.542694 -0.449323 -1.289371
C 0.448841 1.795819 0.495755
H 0.682271 2.738099 -0.040719
O 0.425495 1.701557 1.691391
C -1.529061 0.518129 -0.686211
O -2.455332 0.796551 -1.289811

Structure 71
Co 0.021586 0.113770 -0.169268
H 0.085139 1.093357 -1.286281
C -1.363444 1.165418 0.182009
O -2.217501 1.894883 0.372446
C 1.712188 0.751262 0.034760
O 2.743235 1.218768 0.108879
C -0.878531 -1.209157 -1.558255
H -1.589775 -1.848778 -1.008858
H -1.304281 -0.592357 -2.365504
O 0.353714 -1.409974 -1.484696
C -0.080402 -1.013219 1.289867
O -0.143543 -1.689908 2.195946

Structure 99
Co -0.916240 -0.060069 -0.035128
H -1.587234 -0.083387 -1.355074
H -2.311892 -0.196007 0.556926
C -1.278206 1.657448 -0.280113
O -1.542617 2.742401 -0.488686
C -0.114679 -0.035785 1.607741
O 0.422315 -0.010745 2.604291
C 0.926864 0.062922 -0.919509
H 0.785187 -0.102981 -1.993830
O 1.842871 -0.909887 -0.380120
C 1.892629 1.210338 -0.597677
H 2.218604 1.807659 -1.452670
H 1.588326 1.888117 0.205292
C 2.839508 0.094790 -0.153709
H 3.164451 0.114926 0.893457
H 3.715926 -0.055292 -0.796224
C -0.965090 -1.817644 -0.353114
O -1.052698 -2.919716 -0.601879

Structure T_9
Co -0.000505 -0.037018 0.507912

H 0.715134 0.455372 1.703061
H -0.707861 0.463803 1.704559
H -0.007836 -1.224256 1.383522
C -1.480194 -0.783591 -0.237703
O -2.426405 -1.281747 -0.617109
C 1.470080 -0.801241 -0.237689
O 2.412418 -1.306996 -0.616672
C 0.009603 1.640546 -0.197787
O 0.016145 2.710027 -0.574431

Structure T_{10}

Co -0.278282 0.023145 -0.123924
C 1.698161 0.057790 -0.294923
O 2.511725 0.889705 -0.057988
H -0.144966 -0.052234 -1.603790
C -0.303664 1.840317 -0.068322
O -0.236566 2.972454 -0.079944
C -0.024893 -0.585980 1.588610
O 0.129975 -0.953464 2.653465
C -2.089249 -0.304787 -0.309945
O -3.177723 -0.559653 -0.502628
C 1.832457 -1.332968 -0.829194
H 2.193416 -1.967036 -0.007699
H 2.561246 -1.402673 -1.647693
C 0.376758 -1.631016 -1.182280
H -0.060799 -2.489781 -0.670246
H 0.208028 -1.765653 -2.251544

Structure T_{11}

Co 0.643497 0.000238 0.193979
H 0.654698 0.000927 1.883182
C 0.521680 1.612200 -0.590792
O 0.393642 2.611169 -1.127492
C 0.521834 -1.611571 -0.591022
O 0.393563 -2.610369 -1.128006
C -1.260882 -0.000157 0.437026
O -1.567322 -0.000294 1.657452
H -0.402893 -0.000052 1.994315
C -2.355828 -0.000350 -0.574452
H -2.176715 0.864591 -1.229714
H -2.176705 -0.865613 -1.229291
C -3.764480 -0.000266 -0.014189
H -3.942375 -0.880787 0.608303
H -3.942317 0.880301 0.608252
H -4.497687 -0.000259 -0.824463
C 2.456074 -0.000094 0.319231
O 3.580014 -0.001019 0.477693

Structure T_{12}
Co 0.400581 -0.034166 -0.027307
C 0.812722 1.723620 0.024881
O 1.099522 2.815627 0.159881
C 1.923598 -0.632470 -0.839010
O 2.876667 -0.999486 -1.339642
C -1.450391 0.451829 0.239421
H -1.961266 -0.500216 1.085886
O -2.032677 1.574520 0.558675
H -2.354762 0.379242 1.165755
C -1.620205 -0.218122 -1.116664
H -1.917426 0.582171 -1.801133
H -0.622467 -0.585410 -1.509906
C -2.579605 -1.393812 -1.140636
H -2.253228 -2.181915 -0.453399
H -3.583481 -1.072889 -0.843914
H -2.646130 -1.831445 -2.139986
C 0.432154 -1.114488 1.395666
O 0.483167 -1.736461 2.352592

Structure T_{13}
Co -0.321601 -0.017203 -0.206956
C -2.045133 -0.608237 0.095239
O -3.082352 -0.966975 0.379804
C -0.713120 1.695047 -0.484945
O -0.966734 2.778692 -0.724275
C 0.406571 -1.630456 -0.551393
O 0.839497 -2.633463 -0.855495
C 0.118421 0.075958 1.788556
H -0.274350 0.993592 2.232169
H -0.223144 -0.779978 2.371976
C 1.615697 0.120283 1.534956
H 2.052497 -0.888619 1.528836
H 2.235995 0.733618 2.205123
C 1.651732 0.534140 0.086192
O 2.611909 0.297726 -0.758756
H 2.264240 1.572307 -0.370903
H 1.551000 1.895389 0.203563
H -0.346577 0.009924 -1.724814

Structure T_{14}
Co 0.357182 -0.039240 0.010483
C 1.091993 1.609495 0.095734
O 1.575704 2.627230 0.247221
C 1.759875 -0.829821 -0.855304
O 2.652933 -1.308220 -1.372413

C -1.334256 0.725652 0.556125
H -1.990903 0.092742 1.187776
O -1.681851 1.969516 0.419500
H -1.871427 1.126332 -0.610622
C -1.674985 -0.099400 -1.198055
H -1.951672 0.596447 -2.015712
H -0.689235 -0.479534 -1.560918
C -2.692929 -1.191982 -0.985202
H -2.374411 -1.872967 -0.189911
H -3.663971 -0.777862 -0.696838
H -2.840578 -1.793158 -1.888236
C 0.211618 -1.261092 1.314308
O 0.149513 -1.982228 2.196414

Structure T_{15}
Co -0.597956 0.000041 -0.162785
C -0.391069 1.533777 0.748032
O -0.220880 2.502285 1.332844
C -0.390669 -1.533353 0.748455
O -0.220268 -2.501720 1.333443
C 0.579115 -0.000103 -1.418529
O 1.636216 -0.000291 -1.997823
H 2.521354 -0.000462 -1.147409
C -2.389450 -0.000300 -0.416695
O -3.508279 -0.000539 -0.631031
C 2.513898 -0.000056 0.947542
H 2.161441 0.920650 1.400196
H 2.162039 -0.920991 1.400212
C 3.405330 0.000211 -0.088483
H 3.921004 0.926475 -0.339712
H 3.921737 -0.925713 -0.339489

Structure T_{16}
Co -0.163481 0.026681 -0.305293
H -0.478722 0.260919 -1.916951
C -0.213065 1.631850 0.522674
O -0.203117 2.623705 1.081844
C 0.439676 -1.408534 0.584698
O 0.872410 -2.293094 1.157940
C 1.550451 0.352461 -0.952742
H 0.613674 0.408310 -1.858142
O 2.626917 0.021490 -0.298519
H 3.415325 0.249831 -0.815478
C -1.955394 -0.407305 -0.223207
O -3.054496 -0.683385 -0.285646

Structure T_{17}

Co 0.034572 0.111456 -0.194294
H 0.114342 1.136242 -1.259538
C -1.185792 1.307487 0.297473
O -1.954703 2.102303 0.558789
C 1.781657 0.601932 -0.003215
O 2.849753 0.975827 0.067277
C -1.288428 -0.913178 -1.396493
H -1.872213 -1.603921 -0.758617
H -0.507940 -0.845367 -2.416963
O 0.026395 -1.375194 -1.561447
C -0.089594 -1.108287 1.202957
O -0.168282 -1.830934 2.069972

Structure T_{18}
Co -0.014366 -0.000089 0.144150
C -1.786566 -0.001380 0.558647
O -2.868197 -0.002047 0.900168
C -0.028055 -1.603012 -0.678891
O -0.011471 -2.595398 -1.238366
C 1.873617 0.001827 0.113709
H 2.576738 0.002589 -0.739670
O 2.405724 0.002237 1.247284
C -0.031053 1.602772 -0.678876
O -0.016497 2.594964 -1.238765
H 0.246390 -0.000320 1.812506
H 1.320635 0.000848 1.785014

Structure T_{19}
Co 0.000039 -0.102513 -0.493577
C -0.000097 -1.041067 1.311878
O -0.000288 -0.454242 2.346153
H -0.000006 -2.159180 1.285262
C 1.756114 -0.448023 -0.482530
O 2.866201 -0.684392 -0.529710
C 0.000037 1.654872 0.063621
O 0.000080 2.746761 0.360900
H 0.000106 0.279434 -1.961983
C -1.756051 -0.447974 -0.482673
O -2.866147 -0.684275 -0.529957
H 0.000050 -1.050065 -1.637571

Structure T_{20}
Co -0.797114 0.375935 -0.144938
H -1.049806 0.565336 -1.593513
H -2.131914 1.083453 0.118936
C -0.154525 2.001147 -0.415741
O 0.217845 3.051517 -0.648219

C -0.447600 0.137168 1.635278
O -0.205770 -0.008876 2.732370
C 0.901862 -0.711891 -0.605204
H 1.034740 -0.674194 -1.704636
O 1.017873 -1.869084 -0.019218
C 2.419704 0.225695 -0.197853
H 2.556515 0.894552 -1.042282
H 2.357316 0.691779 0.781954
C 2.912953 -1.076444 -0.267993
H 3.180261 -1.653445 0.609600
H 3.185404 -1.515867 -1.223885
C -1.748455 -1.092105 -0.511324
O -2.394207 -1.978967 -0.796913

The following is the Cartesian coordinates of stereoisomers and conformers of $\mathbf{I_3}$ found by our method:

Structure $\mathbf{I_3}$-1
Co -0.010641 0.057940 -0.240539
C 0.551709 -1.681513 -0.888559
O 0.942460 -2.904134 -1.360201
C 0.193592 -0.564544 1.585984
O 0.325477 -0.969620 2.885682
C 1.838015 0.661006 -0.257061
O 3.128410 1.108774 -0.266603
C -1.853291 -0.554659 -0.240461
O -3.159550 -0.957019 -0.244628
C -0.600322 1.870660 0.426178
H -1.469038 1.760035 1.110665
H 0.225893 2.335815 1.007658
C -0.983883 2.782289 -0.736193
H -1.239757 3.797141 -0.358501
H -1.866583 2.372992 -1.274554
H -0.132509 2.866198 -1.444056

Structure $\mathbf{I_3}$-2
Co 0.126122 -0.023455 0.426297
C -1.369498 -1.267331 0.406419
O -2.451584 -2.101763 0.407267
C 1.349038 -1.508028 0.169338
O 2.234935 -2.536002 0.004906
C 0.009300 0.203578 -1.499576
O -0.078251 0.384253 -2.850724
C 1.653001 1.174691 0.461169
O 2.716414 2.032849 0.497241
C -1.138825 1.560375 0.728758
H -1.261629 1.675768 1.826721
H -0.645349 2.483055 0.353567

C -2.533550 1.476132 0.081309
H -3.067020 2.440222 0.226697
H -3.160046 0.675545 0.540732
H -2.460156 1.287499 -1.011739

Structure I_3-3
Co 0.139975 0.053605 0.440726
C 1.603483 -1.218786 0.455637
O 2.622809 -2.129137 0.501279
C -0.089766 -0.254585 -1.460453
O -0.290270 -0.517978 -2.784211
C 1.415468 1.457250 0.050055
O 2.325995 2.444356 -0.200893
C -1.282488 1.372783 0.436870
O -2.302630 2.281680 0.460960
C -1.203580 -1.432223 0.889315
H -0.685938 -2.415707 0.879830
H -1.536494 -1.253344 1.934592
C -2.452309 -1.537672 -0.009855
H -2.194933 -1.783674 -1.069311
H -3.043233 -0.596348 0.001922
H -3.110790 -2.350232 0.366864

Structure I_3-4
Co 0.006666 0.049784 -0.239230
C -1.896596 0.438436 -0.253531
O -3.227929 0.745366 -0.272479
C -0.120664 -0.558199 1.599989
O -0.200276 -0.949819 2.907397
C 1.908763 -0.344410 -0.247313
O 3.252526 -0.590793 -0.261059
C -0.361266 -1.746008 -0.875811
O -0.618302 -3.005577 -1.338392
C 0.391299 1.920432 0.409123
H -0.459690 2.291956 1.020265
H 1.289179 1.914545 1.064543
C 0.626009 2.873710 -0.759760
H 0.831507 3.895606 -0.374072
H -0.274139 2.915714 -1.410505
H 1.499740 2.540846 -1.360938

Structure I_3-5
Co 0.078595 -0.055463 -0.356877
C 1.222242 1.503138 -0.482084
O 2.032969 2.599045 -0.587301
C 1.647389 -1.197215 -0.422518
O 2.749841 -2.002671 -0.483175

C -1.444312 1.152438 -0.293736
O -2.519176 1.997341 -0.266762
C 0.213120 0.029083 1.577613
O 0.310673 0.091026 2.940632
C -1.079146 -1.737409 -0.253038
H -0.880924 -2.274197 0.697638
H -0.751106 -2.407067 -1.076643
C -2.588851 -1.545201 -0.386200
H -2.828497 -1.020371 -1.334745
H -2.987748 -0.970564 0.477734
H -3.090905 -2.537219 -0.395678

The following is the Cartesian coordinates of stereoisomers and conformers of $\mathbf{I_5}$ found by our method:

Structure $\mathbf{I_5}$-1

Co 0.502512 -0.001641 0.475214
C 0.497235 1.188280 -0.826443
O 0.471042 1.997158 -1.709410
C 0.978461 -1.244769 -0.677954
O 1.295650 -2.103408 -1.451776
C 2.199172 0.350028 0.789307
O 3.351565 0.579977 1.018334
H 0.073857 1.124435 1.537014
H 0.526199 -1.085274 1.635151
C -1.418676 -0.418994 0.158026
O -1.787403 -1.583035 0.222194
C -2.422742 0.668149 -0.145288
H -2.053198 1.633973 0.260930
H -2.517333 0.773148 -1.248341
C -3.799512 0.379954 0.458013
H -3.727336 0.245361 1.558886
H -4.483993 1.229697 0.248904
H -4.236464 -0.538471 0.007965

Structure $\mathbf{I_5}$-2

Co 0.638098 0.398994 -0.526145
C -0.728199 1.483773 -0.789208
O -1.654123 2.220807 -0.973152
C 2.023763 -0.661439 -0.270663
O 2.970538 -1.375423 -0.095294
C 0.789050 0.948918 1.141741
O 0.898914 1.322402 2.275319
H 1.628087 1.572247 -0.984812
H 0.533625 -0.078334 -2.051048
C -0.596159 -1.087380 0.002435
O -0.448982 -2.188498 -0.509123
C -1.727846 -0.893355 0.977263

H -1.646781 -1.651768 1.787871
H -1.663937 0.112446 1.433307
C -3.082278 -1.023566 0.285589
H -3.198403 -2.035931 -0.159022
H -3.894079 -0.868990 1.027759
H -3.187908 -0.258516 -0.513085
